# CIITA-Transduced Glioblastoma Cells Uncover a Rich Repertoire of Clinically Relevant Tumor-Associated HLA-II Antigens

**DOI:** 10.1074/mcp.RA120.002201

**Published:** 2021-01-06

**Authors:** Greta Forlani, Justine Michaux, HuiSong Pak, Florian Huber, Elodie Lauret Marie Joseph, Elise Ramia, Brian J. Stevenson, Michael Linnebacher, Roberto S. Accolla, Michal Bassani-Sternberg

**Affiliations:** 1 Laboratories of General Pathology and Immunology “Giovanna Tosi”, Department of Medicine and Surgery, School of Medicine, University of Insubria, Varese, Italy; 2 Ludwig Cancer Research Center, University of Lausanne, Lausanne, Switzerland; 3 Department of Oncology, Centre Hospitalier Universitaire Vaudois (CHUV), Lausanne, Switzerland; 4 SIB Swiss Institute of Bioinformatics, Lausanne, Switzerland; 5 Department of General Surgery, Molecular Oncology and Immunotherapy, University Medical Center Rostock, Rostock, Germany

**Keywords:** Class II major histocompatibility complex transactivator, Immunopeptidomics, Antigen discovery, Glioblastoma, a.u., arbitrary units, APCs, antigen presenting cells, CIITA, class II major histocompatibility complex transactivator, CTL, cytotoxic T cells, ER, endoplasmic reticulum, ERAAP, ER aminopeptidase-associated with antigen processing, FDR, false discovery rate, GBM, glioblastoma, HCD, higher-energy collision dissociation, HLA, human leukocyte antigen, HLA-I, human leukocyte antigen class I, HLA-II, human leukocyte antigen class II, IFNγ, Interferon gamma, LFQ, label-free quantification, MHC, major histocompatibility complex, PBMCs, peripheral blood mononuclear cells, PSM, peptide spectrum match, SNPs, single nucleotide polymorphisms, SNVs, single nucleotide variants, TAAs, tumor-associated antigens, TAP, transporter-associated with antigen processing, TcR, T cell receptors, TH, T helper cells, WES, whole-exome sequencing

## Abstract

CD4+ T cell responses are crucial for inducing and maintaining effective anticancer immunity, and the identification of human leukocyte antigen class II (HLA-II) cancer-specific epitopes is key to the development of potent cancer immunotherapies. In many tumor types, and especially in glioblastoma (GBM), HLA-II complexes are hardly ever naturally expressed. Hence, little is known about immunogenic HLA-II epitopes in GBM. With stable expression of the class II major histocompatibility complex transactivator (CIITA) coupled to a detailed and sensitive mass spectrometry–based immunopeptidomics analysis, we here uncovered a remarkable breadth of the HLA-ligandome in HROG02, HROG17, and RA GBM cell lines. The effect of CIITA expression on the induction of the HLA-II presentation machinery was striking in each of the three cell lines, and it was significantly higher compared with interferon gamma (IFNɣ) treatment. In total, we identified 16,123 unique HLA-I peptides and 32,690 unique HLA-II peptides. In order to genuinely define the identified peptides as true HLA ligands, we carefully characterized their association with the different HLA allotypes. In addition, we identified 138 and 279 HLA-I and HLA-II ligands, respectively, most of which are novel in GBM, derived from known GBM-associated tumor antigens that have been used as source proteins for a variety of GBM vaccines. Our data further indicate that CIITA-expressing GBM cells acquired an antigen presenting cell-like phenotype as we found that they directly present external proteins as HLA-II ligands. Not only that CIITA-expressing GBM cells are attractive models for antigen discovery endeavors, but also such engineered cells have great therapeutic potential through massive presentation of a diverse antigenic repertoire.

## Highlights

• CIITA-expressing glioblastoma cells express high levels of HLA-II complexes.
• CIITA induces peptide presentation across the HLA-DP, -DQ, and -DR allotypes.
• Many clinically relevant glioblastoma-associated tumor antigens were detected.
• Such cells may have great therapeutic potential in clinical settings.

## Graphical Abstract

**Figure:**
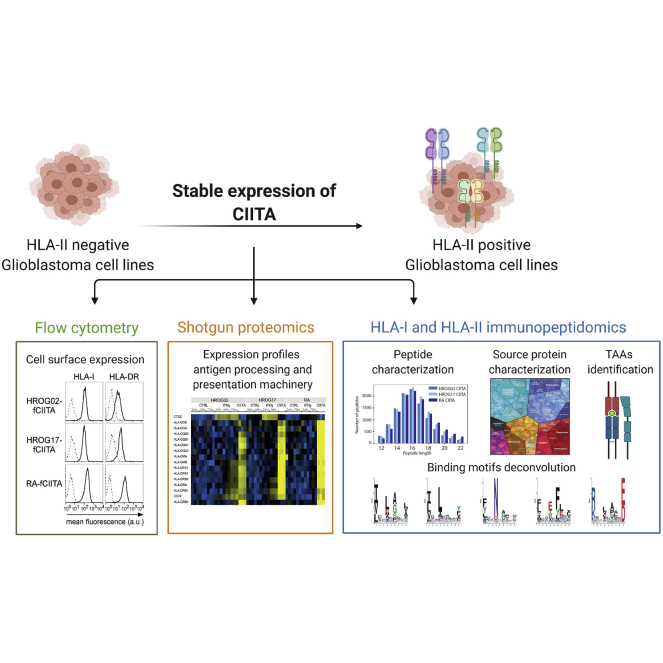

## In Brief

CD4+ T cell responses are crucial for inducing and maintaining effective anticancer immunity; however, in glioblastoma and many solid tumors, HLA-II complexes are hardly ever naturally expressed. Hence, little is known about immunogenic HLA-II epitopes in glioblastoma. With stable expression of the class II major histocompatibility complex transactivator (CIITA) coupled to a detailed immunopeptidomics, we uncovered a remarkable breadth of the HLA-ligandome in three glioblastoma cell lines and identified many cancer-associated ligands with implications for the development of cancer immunotherapies.

Glioblastoma (GBM) is the most aggressive and deadly form of brain cancer in adults, known as a highly immunosuppressive tumor (1). In recent years, in order to overcome barriers in immune recognition, novel immunotherapy approaches aimed at modulating the antigenicity of GBM tumors have been tested, and efforts were directed toward the characterization of the antigenic landscape in GBM through mass spectrometry (MS)-based immunopeptidomics and T cell recognition assays.

T cells directly recognize tumor cells through specific interactions between their T cell receptors (TcR) and antigens presented on the tumor cells as short peptides in complex with the human leukocyte antigens molecules (HLA; known as major histocompatibility complex (MHC) in other organisms). Effective elimination of tumor cells *in vivo* is mediated by CD8+ cytotoxic T cells (CTL) *via* interactions with HLA class I complexes, with help of CD4+ T helper cells (TH) *via* interactions with HLA class II complexes. CD4+ TH cells enhance maturation, proliferation, and functionality of CTL, and the priming and activation of CD4+ TH cells are therefore crucial for inducing and maintaining effective anticancer adaptive immune responses. However, in GBM, like in many other solid tumors, HLA-II molecules are rarely directly expressed on the cancerous cells (2). The common dogma is that tumor antigens induce TH responses when presented on professional antigen-presenting cells APCs (3, 4). Macrophages are a major population of immune cells infiltrating GBM that are involved in removal of cell debris and antigen presentation (5). Similarly, also tumor-infiltrating dendritic cells have been shown to take part in antigen presentation and priming of CTLs with exogenous antigens and to trigger secretion of the inflammatory cytokines interleukin 2 (IL-2) and interferon gamma (IFNɣ) (6, 7). In addition, microglia cells are the main immune cells in the central nervous system; however, the immunosuppressive tumor microenvironment in GBM downregulates HLA expression, and consequently their ability to present antigens is compromised (8). Importantly, because GBM tumors often are HLA-II negative, antigen discovery studies aiming to identify GBM-associated class II antigens resulted in a limited repertoire (9).

Nevertheless, expression of HLA molecules in some solid tumors can be upregulated or induced with IFNɣ. IFNɣ has antiviral, immunoregulatory, and antitumor properties, and it alters transcription of multiple genes responsible for a variety of physiological and cellular responses. Upon IFNɣ treatment, normal and cancer cells increase HLA-I and β2m expression and start expressing genes involved in antigen processing, including the transporter associated with antigen processing (TAP), ER aminopeptidase associated with antigen processing (ERAAP), and subunits of the immunoproteasome (such as MECL1, LMP2, LMP7) (10). IFNɣ-mediated upregulation of HLA-II expression is manifested mainly in professional APCs and to a much lesser extent on tumor cells. Among many other genes, IFNɣ induces the expression of CIITA (11, 12, 13, 14). CIITA regulates the expression of HLA-II genes and also of other key players necessary for HLA-II transport to endosomal compartments and loading of peptides. Among these are the invariant chain that protects spontaneous peptide binding to the groove of HLA-II molecules and human leukocyte antigen DM (HLA-DM) that is involved in the peptide exchange necessary for specific peptide loading of HLA-II molecules (15).

We have shown before that stable expression of CIITA in human tumor cells leads to constitutive functional expression of HLA-II, that endogenous proteins can also be directed to presentation on HLA-II complexes, and that HLA-II expressing cells may directly engage and activate CD4+ TH cells (reviewed in (16)). In mice models, CIITA expressing tumor cells can be efficiently rejected when injected into immunocompetent syngeneic mice (17, 18). Vaccination of mice with tumor cells expressing CIITA led to an immune response against the CIITA-transfected tumor and, most importantly, against the parental tumor (17, 18). We further showed that transfecting CIITA into tumor cells raised their ability to *in vivo* prime naïve CD4+ cells, thus, they serve as APCs (19). CIITA-driven MHC-II-expressing tumor cells have a potential to induce a potent TH immune response through a diverse antigenic repertoire and consequently, to a transformation of the tumor microenvironment from a noninflamed to an inflamed phenotype, associated with infiltration of both CD4+ and CD8+ T cells (20).

MS-based immunopeptidomics is a highly sensitive and accurate antigen discovery approach that supports the development of cancer immunotherapy. Several phase I peptide-based vaccine trials have been reported, where naturally presented tumor-associated antigens were identified by MS in a variety of tumor types, including GBM (21, 22). For example, in patients with newly diagnosed GBM, in the Phase I trial of IMA950 (NCT01222221), patients were vaccinated with a mix of a few HLA-A2 restricted peptides as well as two HLA-II epitopes, derived from shared tumor-associated antigens that have been shown to be immunogenic in GBM (23, 24, 25). Another example is the the GAPVAC-101 (NCT02149225) Phase I trial, where personalized selection of peptide-based actively personalized vaccines (APVAC) was based on HLA-ligandome analyses, whole-exome sequencing (WES), and RNA-seq analyses, for the detection of shared tumor antigens as well as private mutated neoantigens (24, 26). In both trials, the vaccines were able to induce antigen-specific CD8+ and CD4+ T cell responses (24). Furthermore, the importance of CD4+-mediated response in eradicating tumors through vaccination has been highlighted in recent personalized anticancer vaccine trials in melanoma (27, 28). However, likely due to the critical challenges in identifying clinically relevant CD4+ epitopes in the often HLA-II negative GBM tumors (9), the inclusion of CD4+ epitopes in anti-GBM vaccines has been very limited, and consequently, the characterization of antigen-specific CD4+-mediated T responses was lagging behind.

Here we explored the expression of key proteins involved in the HLA-I and HLA-II presentation machineries through proteomics and immunopeptidomics approaches, thereby revealing the antigenic landscape of three glioblastoma cell lines upon stable expression of CIITA. By comparing protein expression profiles of these stably CIITA-expressing cells with cells treated with IFNɣ, we concluded that the cellular machinery responsible for HLA-II presentation can be efficiently upregulated in GBM cells and, more importantly, that it is functional. CIITA-expressing GBM cells express very high levels of HLA-II complexes and naturally present a large repertoire of known HLA-I ligands, but most importantly, of novel HLA-II ligands derived from shared and immunogenic tumor-associated antigens (TAAs). We concluded that CIITA-expressing tumor cells are attractive models for antigen discovery endeavors, especially in HLA-II negative tumors such as GBM. While future work will be required to test the therapeutic potential of each of these antigens, as the antigenicity of CIITA-glioblastoma tumors is greatly enhanced, we propose that vaccination approaches with CIITA-expressing GBM cells could be effective, potentially by sensitizing the tumors to other immunotherapies.

## Experimental Procedures

### Generation of GBM CIITA Cells

The primary culture glioblastoma cell lines HROG02 and HROG17 have been previously described (29). The RA glioblastoma cell line (30) was the generous gift of Dr Pierre Robe, Utrecht University Medical Center, The Netherlands. All GBM cell lines were grown in RPMI 1640 medium (Lonza BioWhittakerTM, Catalog number: BE12-702F) supplemented with 10% heat-inactivated fetal calf serum (FCS) without antibiotics. GBM tumor cells were transfected with 5 μg of flag-CIITA (pcfCIITA) expression vector by using FugeneHD (Promega, catalog number E2311) as described (31). CIITA-transfected GBM cells underwent G418 selection (1 mg/ml for HROG02 and HROG17 and 0.4 mg/ml for RA). HLAII-positive cells were enriched by fluorescence-activated cell sorting with a BD FACS ARIA II cell sorter (Becton- Dickinson, catalog number 95131) and subjected to limiting-dilution cloning. In addition, peripheral blood mononuclear cells (PBMCs) were obtained from HROG02 and HROG17 for NGS analyses (see below).

Informed consent of the participants was obtained following requirements of the institutional review board (approval A 2009-34 by the local ethical commission of the University Medical Center Rostock). The translational research has been approved by the CHUV ethics committee (protocol 2017-00305).

### HLA Typing

Genomic DNA was extracted from HROG02 and HROG17 GBM cells with the commercially available DNeasy Blood & Tissue Kit (Qiagen, Hilden, Germany), following manufacturers’ protocols. In total, 500 ng of gDNA was used to amplify HLA genes by PCR. High-resolution four-digit HLA-I and HLA-II typing was performed in-house using the HLA amplification method with the TruSight HLA v2 Sequencing Panel kit (CareDx) according to the manufacturer’s protocol (Table 1). Sequencing was performed on the Illumina MiniSeq System (Illumina) using a paired end 2 × 150 bp protocol. The data were analyzed with Assign TruSight HLA v2.1 software (CareDx).

**Table 1:** Description of the three GBM samples and their HLA typing

Sample name	HLA-A	HLA-B	HLA-C	HLA-DQA1	HLA-DQB1	HLA-DPA1	HLA-DPB1	HLA-DRB1	HLA-DRB345
HROG02	A:01:01	B:08:01	C:06:02	DQA1∗02:01	DQB1∗02:01	DPA1∗01:03	DPB1∗04:01	DRB1∗03:01	DRB3∗01:01
	A:02:01	B:13:02	C:07:01	DQA1∗05:01	DQB1∗02:02	x	x	DRB1∗07:01	DRB4∗01:03
HROG17	A:11:01	B:14:02	C:01:02	DQA1∗01:01	DQB1∗03:01	DPA1∗01:03	DPB1∗04:01	DRB1∗01:02	x
	A:66:01	B:40:02	C:08:02	DQA1∗05:05	DQB1∗05:01	DPA1∗02:01	DPB1∗11:01	DRB1∗12:01	DRB3∗02:02
RA	A:02:01	B:35:03	C:04:01	DQA1∗03:01	DQB1∗03:02	DPA1∗01:03	DPB1∗04:01	DRB1∗04:01	DRB4∗01:03
	A:24:02	B:51:01	C:14:02	DQA1∗04:01	DQB1∗04:02	DPA1∗02:07	DPB1∗19:01	DRB1∗08:01	x

### IFNɣ Treatment

In total, 5 × 10^5^ GBM cells (HROG02, HROG17, RA) were plated in 10 cm plates and treated with 500 U/ml of IFNɣ (Origene, catalog number TP723162) or with its vehicle. Forty-eight hours after treatment, the cells were collected and analyzed by immunofluorescence and flow cytometry, to assess HLA-I and HLA-II expression, as indicated above. The cell pellet was collected and stored at −80 °C for the subsequent analysis. Three independent experiments for each cell line were performed.

### Flow Cytometry (Cell Surface Expression of HLA-I and HLA-II)

The cell surface expression of HLA class I and HLA class II molecules was assessed by immunofluorescence and flow cytometry (BD FACSAriaTM II Cell Sorter, BD Biosciences). Briefly, the cells were collected, washed with PBS, and then incubated with the anti-HLA antibodies for 30 min on ice. After washing, the cells were incubated with FITC-labeled anti-mouse Ab for 30 min on ice. The following monoclonal antibodies (mAb) were used for HLA staining: anti-HLA class I (A,B,C common) (B9.12.1), anti-HLA class II DR (D1.12), anti-HLA class II DP (B7/21), anti-HLA class II DQ1 (BT/3.4), DQ2 (XIII358.4), and DQ3 (XIV466.2). Anti-DQ mAb used recognized each of the multiple DQ alleles, and all together cover the entire set of described DQ alleles (32). As secondary antibodies, either an FITC-conjugated anti-mouse F(ab)2 (Sigma) or FITC-labeled IgG2a antibodies were used. The data were analyzed using FlowJo 9.5.2 software.

### Generation of Customized Reference Databases for Proteogenomic-Based Neoantigen Identification

DNA was extracted from HROG02 and HROG17 GBM cells and matched PBMCs for exome sequencing with the commercially available DNeasy Blood & Tissue Kit (Qiagen, Hilden, Germany), following manufacturers’ protocols. For exome sequencing, SureSelect Exome V5 library type (Sureselect v5 capture, Agilent Technologies, Santa Clara, CA, USA) and paired end reads were chosen, with at least 100x coverage for the tumor and PBMCs.

For mutation calling and generation of personalized reference databases, we applied the NeoDisc pipeline as previously described (33). Briefly, exome sequence reads were aligned to the Genome Reference Consortium Human Build 37 assembly (GRCh37) with BWA-MEM version 0.7.17 (34). The resulting SAM format was sorted by chromosomal coordinate and converted into a BAM file, then PCR duplicates were flagged, using the Picard AddOrReplaceReadGroups and MarkDuplicates utilities, respectively (from http://broadinstitute.github.io/picard). Various quality metrics were assessed with the Picard MarkDuplicates, CollectAlignmentSummaryMetrics, and CalculateHsMetrics utilities. Following GATK best practices, GATK BaseRecalibrator (within GATK v3.7-0) was used to recalibrate base quality scores (BSQR) prior to variant calling (35, 36). BQSR corrects base quality scores based on an estimation of empirical error frequencies in the alignments. The recalibrated tumor and germline BAM files were then used as input for each of three variant callers: GATK HaplotypeCaller, MuTect v1, and VarScan 2.

The GATK HaplotypeCaller algorithm improves variant calling by incorporating *de novo* assembly of haplotypes in variable regions, thus reducing the overall false-positive variant call rate (35, 36). HaplotypeCaller was run in GVCF mode on each tumor and germline recalibrated BAM file to detect SNV and Indel (insertions/deletions) variants. The resultant gVCF files were combined using GATK GenotypeGVCF to produce raw variant calls for tumor and germline within a single VCF. Subsequent variant quality score recalibration, following GATK best practices, was performed separately for SNVs and Indels using the GATK variant Recalibrator tool to identify high-confidence calls. Variant quality was assessed by the GATK VariantEval tool. Patient-specific SNPs were defined as variants present in both tumor and germline, while variants present only in tumor were defined as somatic mutations.

The MuTect v1 variant calling algorithm predicts somatic mutations based on log odds scores of two Bayesian classifiers (from https://github.com/broadinstitute/mutect). The first classifier identifies nonreference variants in the tumor sample while the second detects whether those variants are tumor-specific. Candidate somatic mutations are then filtered based on read support, for example, by ensuring that supporting reads map to both DNA strands, in order to reduce NGS artifacts. Identified somatic mutations are exported in VCF format.

The VarScan2 algorithm, unlike GATK and MuTect, relies on hard filtering of calls rather than Bayesian statistics (37). This has the advantage of being less sensitive to bias such as extreme read coverage and sample contamination. VarScan 2 filters read based on parameters such as read quality, strand bias, minimum coverage, and variant frequency. The multisample pileup file required for VarScan 2 input was generated with SAMtools (38, 39). VarScan 2 was run using default parameters and generated a VCF containing SNVs and Indels for both somatic mutations and SNPs.

Variant calls from GATK, MuTect v1, and VarScan 2 were combined into a single VCF that contains the union of the variants of all three callers. Ambiguous calls were resolved by a simple majority rule, and if there was no majority, the call was rejected. GATK ReadBackedPhasing was used to retrieve the phasing information of all variants in the combined VCF (35, 36). The functional effect of the variants was annotated by SnpEff, which predicts the effects of variants on genes based on reference databases. To maximize variant annotation, we used annotations from the hg19 (Refseq) and GRCH37.75 (Ensembl) databases (40, 41, 42). This nonredundant, annotated VCF file was used for further genomics and proteogenomics analyses. We used the GENCODE v24 (43) (GRCh37 human reference assembly, downloaded from https://www.gencodegenes.org/human/release_24lift37.html) as the standard reference data set (89,543 entries). We parsed the GENCODE comprehensive gene annotation file, in GFF3 format, to extract genomic coordinate information for every exon. These coordinates were compared with sample-specific variant coordinates to derive nonsynonymous amino acid changes within each protein. This information was used to build a sample-specific fasta file with variants annotated in the fasta header in a format that is compatible with MaxQuant variant search with identifier parse rule >([^∧^∖s]∗) and variation parse rule >[^∧^∖s]+∖s([^∧^∖s]+) as indicated below.

### Sample Preparation for Shotgun Proteomics

Cell pellets of approximately one million cells were resuspended in 8 M Urea (Biochemica, Billingham, UK) and 50 mM ammonium bicarbonate (AMBIC, Sigma-Aldrich) buffer at pH 8. The lysates were sonicated in the Bioruptor instrument (Diagenode, B01020001, Seraing, Belgium) for 15 cycles of 30 s at maximum mA at 4 °C. Subsequently, after centrifugation at 20,000*g* at 4 °C for 30 min, the soluble protein fraction was collected and the protein concentration was determined by a Bradford protein assay. Proteins were reduced with a final concentration of 5 mM DTT (Sigma-Aldrich) at 37 °C for 60 min and then alkylated for 60 min in the dark at room temperature with a final concentration of 15 mM iodoacetamide (IAA, Sigma-Aldrich). The digestion was carried out with a mixture of endoproteinase Lys-C and Trypsin (Trypsin/Lys-c Mix, Promega, Madison, WI). The first step consists of endoproteinase Lys-C digestion for 4 h at 37 °C with a protein-to-enzyme ratio of 50:1 (w/w). Next, the samples were diluted eight times with 50 mM AMBIC to a urea concentration of 1 M. The second step of digestion was performed with trypsin overnight at 37 °C with a substrate-to-enzyme ratio of 50:1 (w/w). After digestion, the samples were acidified with formic acid (FA) and desalted on C18 spin columns (Harvard Apparatus, Holliston, MA).

### Immunoaffinity Purification of HLA Class I and HLA Class II Complexes and Peptide Extraction

For HLA-I and -II immunoaffinity purification, we used a previously described protocol from our group (44, 45). We used the 96-well single-use microplate with glass fiber and 10 μm polypropylene membranes (ref number: 360063, Seahorse Bioscience, North Billerica, MA). Cell lysis was performed at 4 °C for 1 h with PBS buffer solution containing 0.25% sodium deoxycholate (Sigma-Aldrich), 0.2 mM IAA (Sigma-Aldrich), 1 mM EDTA, 1:200 Protease Inhibitors Mixture (Sigma-Aldrich), 1 mM Phenylmethylsulfonylfluoride (Roche, Basel, Switzerland), 1% octyl-beta-D glucopyranoside (Sigma-Alrich). Lysates were cleared by centrifugation at 4 °C at 14,200 rpm for 30 min with a table-top centrifuge (Eppendorf Centrifuge, Hamburg, Germany). Anti-pan HLA-I (HB95) and HLA-II (HB245) antibodies cross-linked to protein-A sepharose 4B beads (Invitrogen, Carlsbad, California) were loaded on their respective plates at a final bead volume of 100 μl. The lysates were loaded first through the HLA-I affinity plate and then through the HLA-II affinity plate by gravity at 4 °C. Using the Waters Positive Pressure-96 Processor (Waters, Milford, MA), each plate was washed four times with 2 ml of 150 mM sodium chloride (NaCl) (Carlo-Erba, Val de Reuil, France) in 20 mM Tris-HCl pH 8, four times with 2 ml of 400 mM NaCl in 20 mM Tris-HCl pH 8, four times with 2 ml of 150 mM NaCl in 20 mM Tris-HCl pH 8, and finally, twice with 2 ml of 20 mM Tris-HCl pH 8.

Two Sep-Pak tC18 100 mg Sorbent 96-well plates (Waters) were required for the purification and concentration of HLA-I and HLA-II peptides. Each Sep-Pak tC18 plate was handled separately and equilibrated with 80% acetonitrile (ACN) in 0.1% TFA and washed with 0.1% TFA. Each affinity plate was stacked on top of a Sep-Pak tC18 plate, and the HLA complexes were eluted with 500 μl of 1% TFA. The Sep-Pak tC18 wells were washed with 2 ml of 0.1% TFA. Thereafter, the HLA peptides were eluted with 500 μl of 32% ACN in 0.1% TFA. The recovered HLA-I and -II peptide samples were transferred separately into Eppendorf tubes, dried using vacuum centrifugation (Concentrator plus Eppendorf), and stored at 20 °C.

### MS Analyses

Samples were acquired using the nanoflow UHPLC Easy-nLC 1200 (ThermoFisher Scientific, LC140) coupled online to a Q Exactive HF or a Q Exactive HF-X Orbitrap mass spectrometer (Thermo Fischer Scientific) with a nanoelectrospray ion source (Nanospray Flex Ion Sources, ThermoFisher Scientific, USA)). We packed the uncoated PicoTip 8 μm tip opening (New Objective, USA) with 75 μm i.d. and 450 mm long analytical columns with ReproSil-Pur C18 (1.9 um particles, 120 Å pore size, Dr Maisch GmbH, Ammerbuch, Germany). Mounted analytical columns were kept at 50 °C using a column oven (Sonation, PRSO V1, Baden Wurttemberg, Germany).

HLA-I and HLA-II peptide samples were resuspended in 12 μl of 0.1% FA/2% ACN, and 3 μl of each sample was placed in the UHPLC autosampler. Peptides were then separated using a linear gradient of solvent A (0.1%FA) and B (0.1% FA/85% B). HLA-I peptides were separated from 2 to 35% B during a total elution of 120 min. HLA-II peptides were separated from 2 to 45% B during a total elution of 90 min. Data was acquired with data-dependent “top10” method, which isolates within a 1.2 *m/z* window the ten most abundant precursor ions and fragments them by higher-energy collision dissociation (HCD) at normalized collision energy of 27%. For MS1 scan, the mass spectrometer was operated to a scan range from 300 to 1650 *m/z* with a resolution of 60,000 (200 *m/z*), a maximum injection time of 20 ms (80 ms for HF-X), and AGC target value of 3e6 ions. For MS/MS, fragment ions were acquired at a resultion of 15,000 (200 *m/z*) with a maximum injection of 120 ms and AGC target value of 1e5 (2e5 for HF-X). The peptide match option was disabled. For HLA-I peptidomics, in case of assigned precursor ion charge states of four and above, no fragmentation was performed. For HLA-II peptidomics, in case of assigned precursor ion charge states of one, and from six and above, no fragmentation was performed. The dynamic exclusion of precursor ions from further selection was set for 20 s.

For shotgun proteomics, tryptic peptides were separated through a 270 min gradient (mainly from 2 to 60% B), and data-dependent “top15” method was used, which isolates within a 1.4 *m/z* window the 15 most abundant precursor ions and fragments them by HCD at normalized collision energy of 27%. For MS1 scan, the mass spectrometer was operated to a scan range from 300 to 1650 *m/z* with a resolution of 60,000 (200 *m/z*), a maximum injection time of 20 ms (80 ms for HF-X), and AGC target value of 3e6 ions. For MS/MS, AGC target values of 1e5 were used with a maximum injection time 25 ms (80 ms for HF-X) at resolution of 15,000 (200 *m/z*). In case of unassigned precursor ion charge states or a charge state of one, no fragmentation was performed and the peptide match option was disabled. The dynamic exclusion of precursor ions from further selection was set at 30.

### Experimental Design

A detailed description of the immunopeptidomics class I, II experiments, including RAW MS file names, assignment of replicates, and cell number used per experiment are provided in supplemental Table S1.

### Identification of Peptides and Proteins

We employed the MaxQuant computational platform version 1.6.10.43 (46) to search the peak lists against the UniProt databases (Human 20,362 entries, May 2020) and a file containing 246 frequently observed contaminants. For proteomics, the default settings were used. Enzyme specificity was set to “Trypsin/P,” and maximum two missed cleavages were allowed per peptide. Methionine oxidation and Protein N-term acetylation were set as a variable modifications, and a fixed modification of cysteine carbamidomethylation was set. The second peptide identification option in Andromeda was enabled. A peptide spectrum match (PSM) false discovery rate (FDR) of 0.01 and protein FDR of 0.01 were set. The initial allowed mass deviation of the precursor ion was set to 6 ppm, and the maximum fragment mass deviation was set to 20 ppm. “Match between runs” and “label-free quantification” (LFQ) modules were enabled with the default parameters (47). For immunopeptidomics, the default settings were used except the following parameters: enzyme specificity was set to ”unspecific,” methionine oxidation and Protein N-term acetylation were set as a variable modifications, and no fixed modification was set, PSM FDR was set to 0.01 with no protein FDR. The initial allowed mass deviation of the precursor ion was set to 6 ppm, and the maximum fragment mass deviation was set to 20 ppm. “Match between runs” was enabled separately for each cell line and for HLA-I and HLA-II peptide samples separately.

For neoantigen identification in proteomics and immunopeptidomics data, we used MaxQuant version 1.5.9.4 with similar settings as above, except that variants were searched from the personalized fasta file (mentioned above).

Data visualization and statistical analyses were performed using the Perseus computational platform version 1.6.6.0 (48). For proteomics data sets, we used LFQ values from “ProteinGroups” MaxQuant output table of the search of tryptic pepitdes. Reverse, contaminants and proteins only identified by site or with a single peptide were filtered, Intensities were log2 transformed. The table was filtered to include proteins with three valid LFQ intensity values in at least one group per cell line and condition (supplemental Table S2). Missing values were imputed with standard parameters, and values were z-scored for visualization purposes. Volcano plots summarizing two-sided Student’s *t*-test results were generated, and points outside the lines indicate significantly upregulated or downregulated proteins (permutation-based FDR = 1%. S0 = 1). Annotations were imported from KEGG, and 2D annotation enrichment module in Perseus was used on Student’s *t*-test difference with Benjamini–Hochberg FDR of 0.02 FDR. For immunopeptidomics data sets, we used the “Peptides” output table. Peptides matching to reverse and contaminants sequences were removed and intensities were log2 transformed. To enrich for high-probability HLA ligands, excluding contaminant peptides from downstream analyses, HLA peptides of 8 to 12 a.a and of 12 to 22 a.a were retained for HLA-I and HLA-II immunopeptidomic data sets, respectively (supplemental Tables S3 and S4).

### Clustering of HLA Peptides and Prediction of Binding to HLA

MixMHCpred (v.2.0.2) (49,50) was used to predict binding of peptides on patients’ HLA class I alleles, and MixMHC2pred (v.1.0) (51) was used to predict binding of peptides on patients’ HLA class II alleles (supplemental Table S5). HLA-DPA1/DPB1 and HLA-DQA1/DQB1 pairs were assigned for each patient based on their availability in MixMHC2pred. For each peptide, the HLA allele with the lowest rank was assigned as binder. Peptides with multiple binding alleles assigned were removed from the analysis. Some of the HLA class II alleles were not available for the prediction analysis, leading to a miss assignment of HLA class II alleles to some of the HLA class II peptides. Those peptides will therefore have an artificially higher binding rank. For the analysis of the distribution of HLA binding peptides, only peptides with rank ≤2 were selected. Peptides assigned to the same HLA allele were grouped together. Analysis was performed as a mix of bash scripts and python (v.3.5.2) scripts. Histograms and barplots were drawn with Matplotlib (v.2.2.3). Violinplots were drawn with seaborn (v.0.9.0). Statistical annotation on plots was done using statannot (v.0.2.3).

HLA binding motif deconvolution of the HLA-II peptides (12–22 amino acids in length) was performed using MoDec (v.1.1) (51) with the following parameters: –L 9–pepLmin 12–nruns 20–specInits–makeReport–Kmin 1–Kmax 9. Binding motif deconvolution of 9 mer HLA-I peptides was performed using MixMHCp 2.1 with the default settings. Upon completion of Modec and MixMHCp 2.1, deconvoluted motifs were analyzed manualy and assigned to patient HLA.

### HLA Sampling–Related Gene Ontology (GO) Enrichment Analysis and Tree-Map Visualization

The GO enrichment analysis of the source proteins of the presented HLA peptides was performed on the DAVID functional annotation tool website (52). All human proteins were taken as a background and compared with the different source protein lists based on the biological process classification and KEGG pathways. A statistical overrepresentation test was performed and the resulting *p*-values were corrected for multiple testing and a *p*-value threshold of 0.05 was applied. For visualizing of the protein lists, we used the Proteomaps tree-map visualization tool (53) that shows quantitative composition of proteomes that are arranged in multiple levels. Functionally related proteins according to a KEGG hierarchy tree are arranged in adjacent and similarly colored regions, and on higher levels, similar proteins are grouped into regions. As a pseudo-quantitative score, we used the average number of assigned HLA-I or HLA-II peptides per source protein per cell line (average of all replicates per cell line). On the lowest level, each protein is represented by a polygon, whose area reflects the number of peptides weighted by protein size. We used the same source protein lists and included average unique and razor peptide counts from the MaxQuant “ProteinGroups” table per sample.

## Results

### Transfection of CIITA Leads to Cell Surface Expression of HLA-II in GBM Cells

**Fig. 1:**
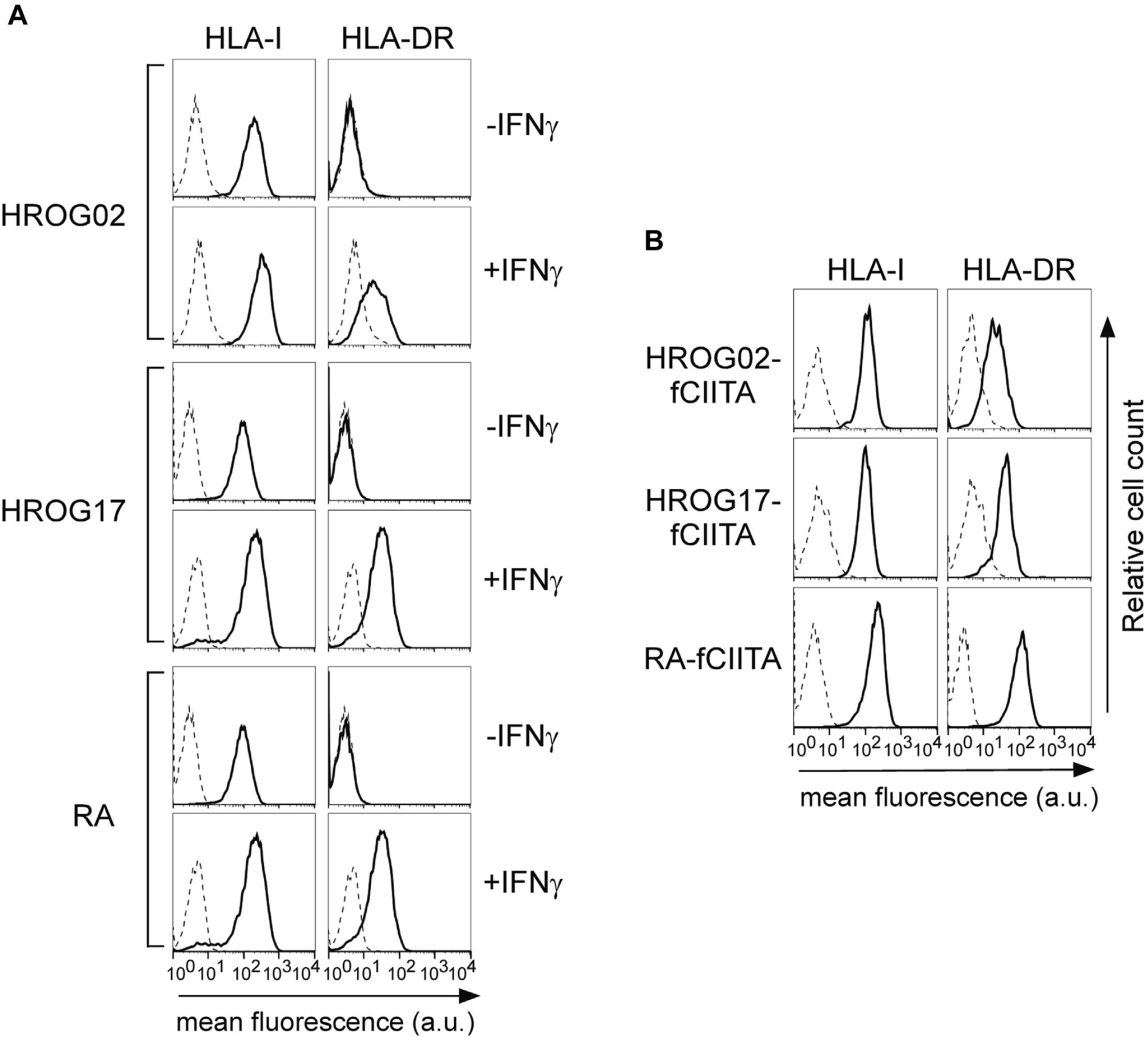
**Expression of HLA class I and HLA class II DR cell surface molecules in HROG02, HROG17, and RA GBM cells after treatment with IFNγ or after stable expression of CIITA.***A*, HLA-I and HLA HLA-DR cell surface expression was assessed by immunofluorescence and FACS analysis in HROG02, HROG17, and RA GBM cells treated for 48 h or not treated with IFNγ (+IFNγ or - IFNγ, respectively). *B*, the stable expression of CIITA in HROG02, HROG17, and RA GBM cells induces HLA-DR expression (HROG02-fCIITA, HROG17-fCIITA, and RA-fCIITA). HLA-I and HLA-DR cell surface expression was assessed as above. Histograms represent fluorescence profiles of the cells indicated on the top incubated with specific anti HLA-I (B9.12.1) or HLA-DR (D1.12) mAbs (*solid line*) followed by incubation with FITC-conjugated F(ab)2 anti-mouse antibody as second reagent. Controls (*dashed line*) are cells incubated with the second reagent only. Mean fluorescence values are expressed in the abscissa as arbitrary unit (a.u.). One representative experiment out of three independent experiments is shown for *A*–*B*, respectively.

In order to study the effect of CIITA on the HLA ligandome of GBM cells, we first characterized cell surface expression of HLA-I and HLA-II with flow cytometry in three patient-derived primary GBM cell lines: HROG02, HROG17, and RA. While HROG02, HROG17, and RA cells express HLA-I, they do not express HLA-DR, HLA-DP, and HLA-DQ molecules (supplemental Fig. S1). However, upon 48 h of *in vitro* stimulation with IFNɣ, cell surface HLA-II expression is induced and detected in each of the three lines (Fig. 1*A*, HLA-DR). Moreover, HLA-I expression is enhanced by IFNɣ (Fig. 1*A*). This result indicates that the cellular machinery responsible for HLA class II antigen processing and presentation is functional in these GBM cells. Indeed, stably transfecting the cells with a vector encoding for CIITA led to an even higher expression level of HLA-DR compared with IFNɣ treatment, in each of the three investigated GBM cell line subclones, named HROG02-CIITA, HROG17-CIITA, and RA-CIITA (Fig. 1*B*).

### Superior Expression of the HLA-II Antigen Processing and Presentation Machinery in GBM Cells Upon Stable Expression of CIITA Than with IFNɣ Treatment

**Fig. 2:**
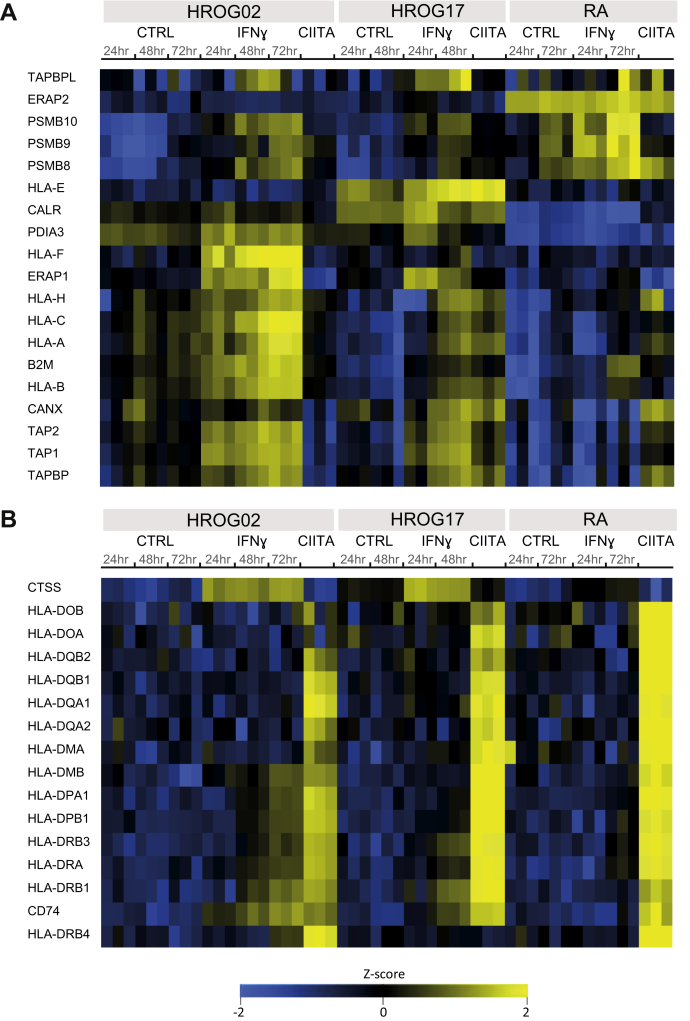
**Enhanced expression of HLA-I (*A*) and HLA-II (*B*) presentation machineries upon IFNγ treatment and stable expression of CIITA, respectively, in HROG02, HROG17, and RA cells.** Heat maps of z-scores Log2 transformed label-free quantification (LFQ) values defined by shotgun proteomic analyses of three biological replicates per condition, following imputation of missing values.

We first characterized the overall effect of stable expression of CIITA on GBM cells and compared it with treatment with IFNɣ. Through shotgun proteomics analyses, we quantified the expression of 6245 proteins (supplemental Table S2), and specifically focused on key proteins involved in HLA-I (Fig. 2*A*) and HLA-II (Fig. 2*B*) antigen processing and presentation, as well as other IFNɣ-induced genes (supplemental Fig. S2). IFNɣ treatment led to a significant increase in expression of the HLA molecules, β2m, TAP1/2, and the components of the antigen-loading complex, such as TAPBP, TAPBPL, CANX, and CALR, in HROG02 and HROG17 cells, and to a lesser extent in RA cells. Upregulation of subunits of the immunoproteasome (PSMB8/9/10) was however more dominant in RA cells compared with HROG02 and HROG17 cells (Fig. 2). The stable expression of CIITA did not induce a significant increase in expression of proteins involved in HLA-I presentation (Figs. 1*B* and 2 and supplemental Fig. S2).

**Fig. 3:**
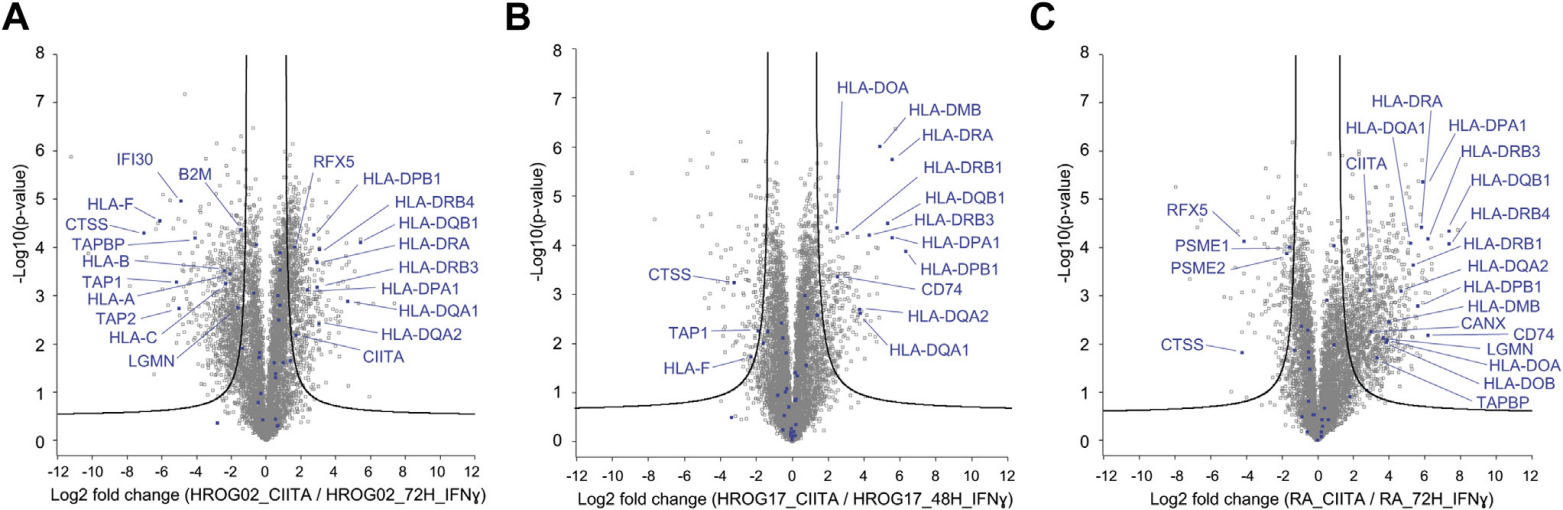
**Stable expression of CIITA leads to higher upregulation of the HLA-II presentation machinery-related proteins than upon IFNγ treatment in HROG02 (*A*), HROG17 (*B*), and RA (*C*) cells.** Volcano plots summarizing two-sided Student’s *t*-test results of three biological replicates per condition. Points outside the lines indicate significantly upregulated or downregulated proteins (permutation-based FDR = 1%. S0 = 1). Proteins related to the “antigen processing and presentation” pathway at the Kyoto Encyclopedia of Genes and Genomes (KEGG) database are highlighted in *blue* and those that are significant are named.

Treatment with IFNɣ did not induce a detectable CIITA expression (supplemental Table S2), as this protein is a transcription factor that is expressed at a very low level. However, in HROG02-CIITA and RA-CIITA cells, the expression of CIITA was high enough to be detected (supplemental Table S2). Overall, the effect of CIITA expression on the induction of the HLA-II presentation machinery was striking in each of the three cell lines, and it was significantly higher compared with the IFNɣ-treated cells (Figs. 2 and 3 and supplemental Fig. S3*A*). For example, we observed that even in the case of HROG02 cell line that was the most IFNɣ-responsive cell line of the three (as seen in Fig. 2), the expression of HLA-DR, HLA-DP, and HLA-DQ was higher in HROG02-CIITA cells than upon IFNɣ treatment (two-sided Student’s t permutation-based FDR = 1%). As expected, we found that the expression of CTSS was not affected by the stable expression of CIITA, but it was induced by IFNɣ treatment, and the other way around, the expression of HLA-DOA and HLA-DOB was not induced by IFNɣ but was higher in CIITA-expressing cells than in the control cells. Interestingly, very low expression levels of HLA-DQA2 and HLA-DQB2, which are paralogous to HLA-DQA1 and HLA-DQB1, respectively, were detected in the CIITA-expressing cells (supplemental Fig. S3*A*). Only limited information is available on the regulation of their expression and on their possible cellular function (54), and more research is needed to define if these are biologically relevant.

Furthermore, 2D annotation enrichment analysis revealed that, for example, in HROG02 cells, immune-related proteins were similarly significantly upregulated in HROG02-CIITA cells as well as upon IFNɣ treatment, while proteins related to cell cycle and DNA replication were significantly downregulated only in IFNɣ-treated cells and were not affected in HROG02-CIITA cells (supplemental Fig. S3*B*). Indeed, in addition to enhancing antigen presentation, the antiproliferative activity of IFNɣ may induce autophagy or apoptosis, resulting in growth inhibition or cell death.

### CIITA-Expressing GBM Cells Present Rich HLA-I and HLA-II Immunopeptidomes Across Allotypes

Next, we applied our high-throughput immunopeptidomics sample preparation approach (44, 45) and purified sequentially HLA-I and -II binding peptides from multiple replicates of HROG02, HROG17, RA cells and from HROG02-CIITA, HROG17-CIITA, RA-CIITA cells. We first characterized the HLA-I immunopeptidome to assure that CIITA is not negatively affecting this pathway. We measured the peptides with LC-MS/MS in technical replicates (supplemental Table S1) and identified in total 16,123 unique HLA-I peptides of 8 to 12 amino acids in length (supplemental Table S3). Among them, 2775, 4143, and 2367 HLA-I peptides were detected in HROG02, HROG17, and RA cells, respectively, and 5113, 6383, and 2547 HROG02-CIITA, HROG17-CIITA, and RA-CIITA cells, respectively (supplemental Fig. S4). The observed increase in the number of identified peptide is likely related to the higher number of CIITA-expressing cells that were used for the analyses (supplemental Table S1), and not to a direct effect of CIITA, as no major differences in expression of the HLA-I presentation machinery were detected in the proteomics analysis (Fig. 2). Overall, the length distribution of the eluted HLA-I peptides was typical, with most of the peptides being 9 mers (supplemental Fig. S5, *A* and *B*). In addition, we applied HLA motif deconvolution and HLA-binding prediction tool that provide complementary validation. On average, 98% of the peptides were predicted to bind the respective HLA allotypes (rank ≤ 2%; MixMHCpred (49, 50)) in both the parental lines and the CIITA-expressing cells (supplemental Fig. S5*C*, and supplemental Table S5). No obvious differences were observed in the distribution of peptides-HLA specificities based on the prediction results (supplemental Fig. S5*D*) as well as by unbiased deconvolution of the 9 mer peptides that revealed the expected HLA-I binding motifs (supplemental Fig. S5, *E*–*G*) (49, 50).

**Fig. 4:**
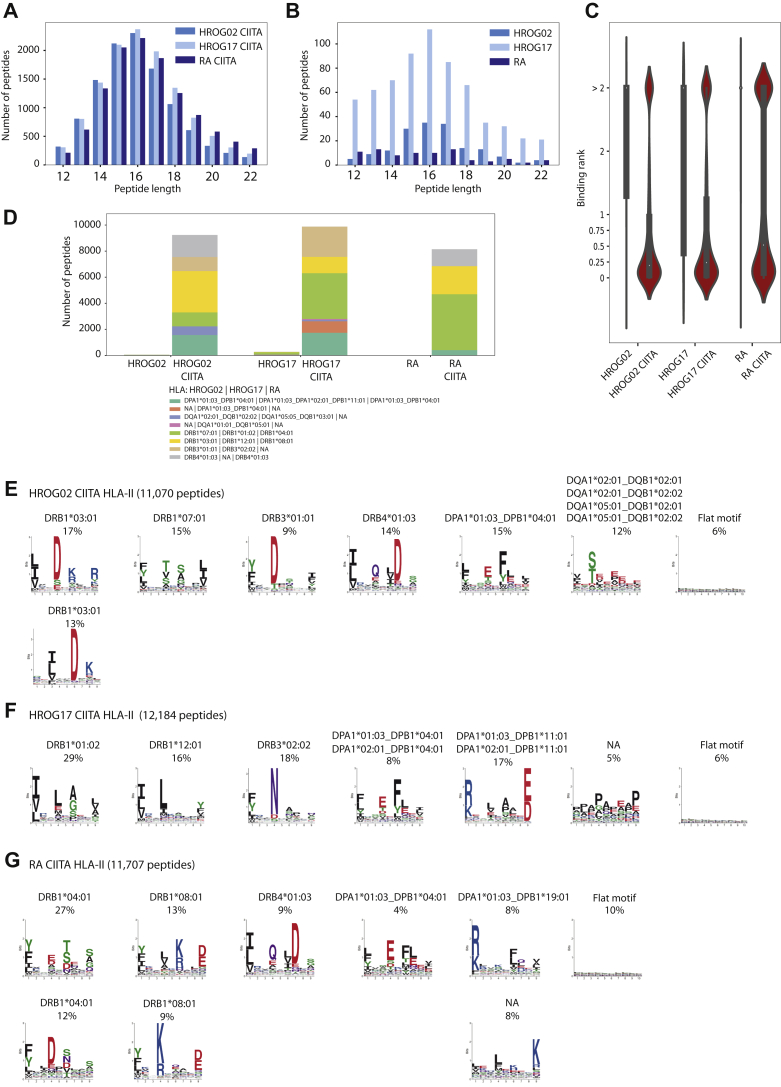
**Characterization of MS-identified HLA-II peptides.** Length distribution of HLA-II peptides identified in HROG02-CIITA, HROG17-CIITA, and RA-CIITA (*A*) and HROG02, HROG17, and RA (*B*) cells. Violin plots show the distribution of the predicted percentage binding ranks to the respective HLA class II alleles expressed in the different samples computed by MixMHC2pr ed (*C*). Distribution of predicted binders (rank ≤ 2%) to the respective alleles in each of the samples (*D*). Motif deconvolution of all 12 to 22 a.a HLA-II peptides identified in the HLA-II ligandome of HROG02-CIITA (*E*) HROG17-CIITA (*F*), and RA-CIITA (*G*) samples. Sample name and number of peptides are indicated. Numbers above each motif indicate the percentage of peptides assigned to that motif. In case of observed multiple specificity or redundancy, motifs are indicated accordingly. NA, not assigned.

We similarly measured by MS the eluted HLA-II peptides and identified in total 32,690 unique HLA-II peptides that were 12 to 22 amino acids in length (supplemental Fig. S4 and supplemental Table S4). There is little data on HLA-II presentation in GBM cells, and in order to genuinely define the identified peptides as true HLA-II ligands, we carefully characterized their association with the different HLA-II allotypes. As GBM cells do not naturally express HLA-II molecules, only 165, 651, and 83 peptides were identified in HROG02, HROG17, and RA cells, respectively. The majority of them were not predicted to bind the respective HLA-II allotypes (rank ≤ 2%; MixMHCpred, Fig. 4, *A*–*C* and supplemental Table S5), suggesting that a background level of potential contaminants contributed to these identifications. However, a 70-fold increase, on average, in the number of HLA-II peptides was detected in HROG02-CIITA, HROG17-CIITA, and RA-CIITA cells, where 11,070, 12,183, and 11,707 unique peptides were identified, respectively. The length distribution of the peptides was found to be typical for HLA-II binding peptides, with an average length of 15 amino acids (Fig. 4, *A* and *B*). In HROG02-CIITA and HROG17-CIITA cells, 83% and 81% of the peptides were predicted to bind the respective HLA-II allotypes expressed in the cells with MixMHC2pred (51), while in RA-CIITA, 70% of the peptides were predicted as binders (Fig. 4*C* and supplemental Table S5).

Specifically for HROG02-CIITA, six HLA-II allotype-combinations are covered by MixMHC2pred, including DRB1∗03:01, DRB1∗07:01, DRB3∗01:01, DRB4:01:03, DPA1∗01:03_DPB1∗04:01, and DQA1∗02:01_DQB1∗02:02, while DQA1∗05:01_ DQB1∗02:01, DQA1∗02:01_DQB1∗02:01, and DQA1∗05:01_DQB1∗02:02 are not covered. Based on the prediction scores, 83% of the identified peptides were predicted to bind (rank ≤ 2%) any of the above six allotypes (Fig. 4*D*). Independent motif deconvolution analysis with MoDec (51) similarly revealed several motifs that showed high similarity to the known binding motifs of the abovementioned alleles (Fig. 4*E* and supplemental Fig. S6) (55). In addition, the proportion of the assigned peptides to each motif is similar in the two independent approaches. No additional new motifs could be resolved even when increasing the number of clusters in MoDec to nine possible clusters. As the peptide binding motifs of the additional HLA-DQ combinations, DQA1∗05:01_DQB1∗02:01, DQA1∗02:01_DQB1∗02:01, and DQA1∗05:01_DQB1∗02:02 are similar and redundant with DQA1∗02:01_DQB1∗02:02 (55), this motif could potentially be assigned to all the four HLA-DQ combinations. When such redundant motifs co-occur in a sample, unbiased clustering of pooled ligandomics data is typically not capable of resolving them, and binding predictors will output highly similar prediction scores in such cases.

For HROG17-CIITA, seven HLA-II allotype combinations are covered by MixMHC2pred, including DRB1∗01:02, DRB1∗12:01, DRB3∗02:02, DPA1∗01:03_DPB1∗04:01, DPA1∗01:03_DPA1∗02:01_DPB1∗11:01 (DPA1∗02:01 only appears in samples also containing DPA1∗01:03 and hence the two alleles are linked for prediction purposes, see MoDec for more information (51)), DQA1∗05:05_DQB1∗03:01, and DQA1∗01:01_DQB1∗05:01, while DPA1∗02:01_DPB1∗04:01, DQA1∗05:05_ DQB1∗05:01, and DQA1∗01:01_ DQB1∗03:01 are not covered. In total, 81% of the identified peptides were predicted to bind any of the above predictable allotypes (Fig. 4*D* and supplemental Table S5). Only a small fraction of 4% of the peptides in HROG17-CIITA sample were predicted to bind DQA1∗05:05_DQB1∗03:01 and DQA1∗01:01_DQB1∗05:01. Similarly, five distinct motifs were identified in the independent motif deconvolution approach that were similar to known motifs of the DR and DP alleles expressed in HROG17-CIITA cells (55, 56) and no additional distinct motifs were found to fit the DQ alleles (supplemental Fig. S6) by increasing the number of clusters to nine possible clusters. In the case of HROG17-CIITA, mainly HLA-DR and HLA-DP complexes are presented in the ligandome, which is in agreement with their expression level and the relatively lower expression level of HLA-DQ as detected by shotgun proteomics (supplemental Fig. S3*A*).

For RA-CIITA, DRB1∗04:01, DRB1∗08:01, DRB4∗01:03, and DPA1∗01:03_DPB1∗04:01 are covered in MixMHC2pred, and accordingly, 70% of the identified peptides were predicted to be binders (supplemental Table S5). By clustering the peptides into eight groups, the motif deconvolution approach optimally revealed distinct motifs matching the binding specificity of these abovementioned predictable allotypes as well as two additional minor motifs comprising each 8% of the peptides, where the first resemble the binding specificity of HLA-DPA1∗01:03_DP1∗19:01 (supplemental Fig. S6), and the second seem to be its mirror image, as was reported before by van Balen *et al.* (56) for HLA-DPA1∗02:02_DPB1∗05:01. Based on similarity to known motifs, none of the resolved motifs matches the specificities of the HLA-DQ alleles expressed in RA-CIITA cell, suggesting again minor contribution of HLA-DQ to the ligandome (supplemental Fig. S6). As precise information is still missing about possible binding in reverse orientation and about the motifs of HLA-DPA1∗02:07_DPB1∗04:01 and HLA-DPA1∗02:07_DPB1∗19:01 pairing combinations, more specific work is needed to resolve them (Fig. 4*G*. and supplemental Fig. S6).

### Sampling of Cellular Proteins for HLA-I and HLA-II Presentation in CIITA-Transduced GBM Cells

**Fig. 5:**
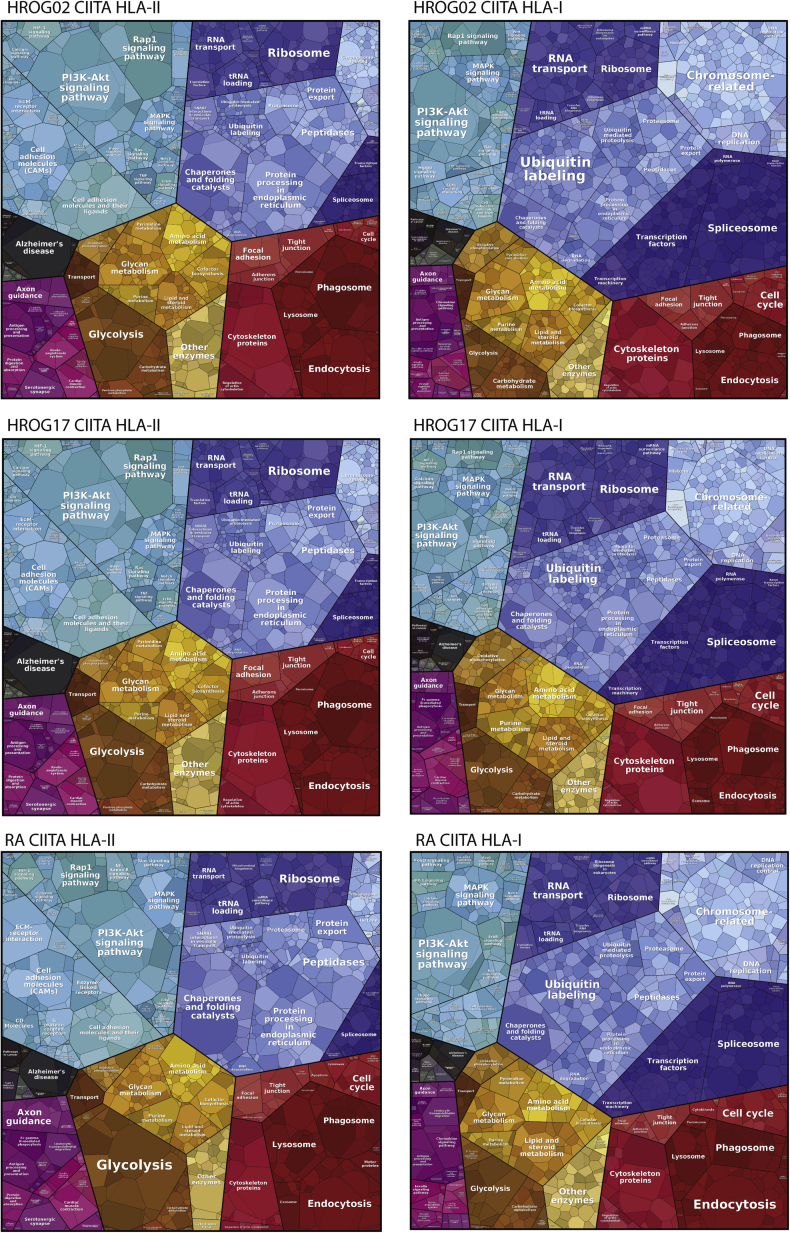
**Proteomaps visualization of the level of presentation of source proteins presented on HLA-I and on HLA-II complexes, in HROG02-CIITA, HROG17-CIITA, and RA-CIITA cells.** The maps show quantitative composition of the presented proteomes that are arranged in multiple levels. Each protein is represented by a polygon, whose area reflects the number of peptides weighted by protein size. Proteins are classified according to their cellular function.

Next, we explored the source proteins that are presented on HLA-II and HLA-I complexes in the three CIITA-expressing GBM cell lines. We further compared them with those presented on cells naturally expressing HLA-II complexes, as B cells and T cells (data obtained from (44)). We compared the characteristics of the source proteins in terms of GO annotation enrichment of biological process, molecular functions, cellular compartments, and KEGG pathways (supplemental Table S6), and we further visualized the data with the Proteomaps tool, which is based on the KEGG protein annotation (source data is available in supplemental Table S7). The Proteomaps shown in Figure 5 revealed overall high similarity in the three CIITA-expressing GBM cell lines between the sampling of the proteome to the HLA-I and HLA-II peptidomes, respectively, and importantly, they were comparable to Proteomaps of B cells and T cells–derived immunopeptidomes (supplemental Fig. S7). Consistent with results from a previous study (57), we found that the source proteins of HLA-II peptides are commonly derived from proteins located in the vesicular compartments and that are involved in particular pathways and cellular processes such as signal transduction and signaling molecules, lysosomes, phagosomes, and cell adhesion (GO annotation enrichment *p*-values are reported in supplemental Table S6). In contrary, the source proteins involved in transcription, DNA maintenance, spliceosome, cell cycle, and transcription factors are less often presented on HLA-II, but are frequently presented and enriched on HLA-I molecules. Furthermore, as expected, cell-type-specific expression of particular proteins would lead to differences in the maps. For examples, tumor-associated antigens such as Chondroitin sulfate proteoglycan 4 (CSPG4) and Tenascin (TNC), also known as Glioma-associated-extracellular matrix antigen, were found only in the GBM cells, while Transferrin receptor protein 1 (TRFC) that is a mediator of iron uptake necessary for, and positively regulates, T and B cell proliferation was highly presented in T and B cells.

Collectively, these results indicate that upon CIITA expression in GBM, HLA-II presentation machinery is functional and capable of sampling the cellular proteome for presentation similar to other cells that naturally express HLA-II. Furthermore, CIITA expression in GBM cells seems to not interfere with sampling of the proteome for HLA-I presentation.

### CIITA Expressing GBM Cells Present HLA-II Peptides Derived from Exogenous Bovine Proteins

**Fig. 6:**
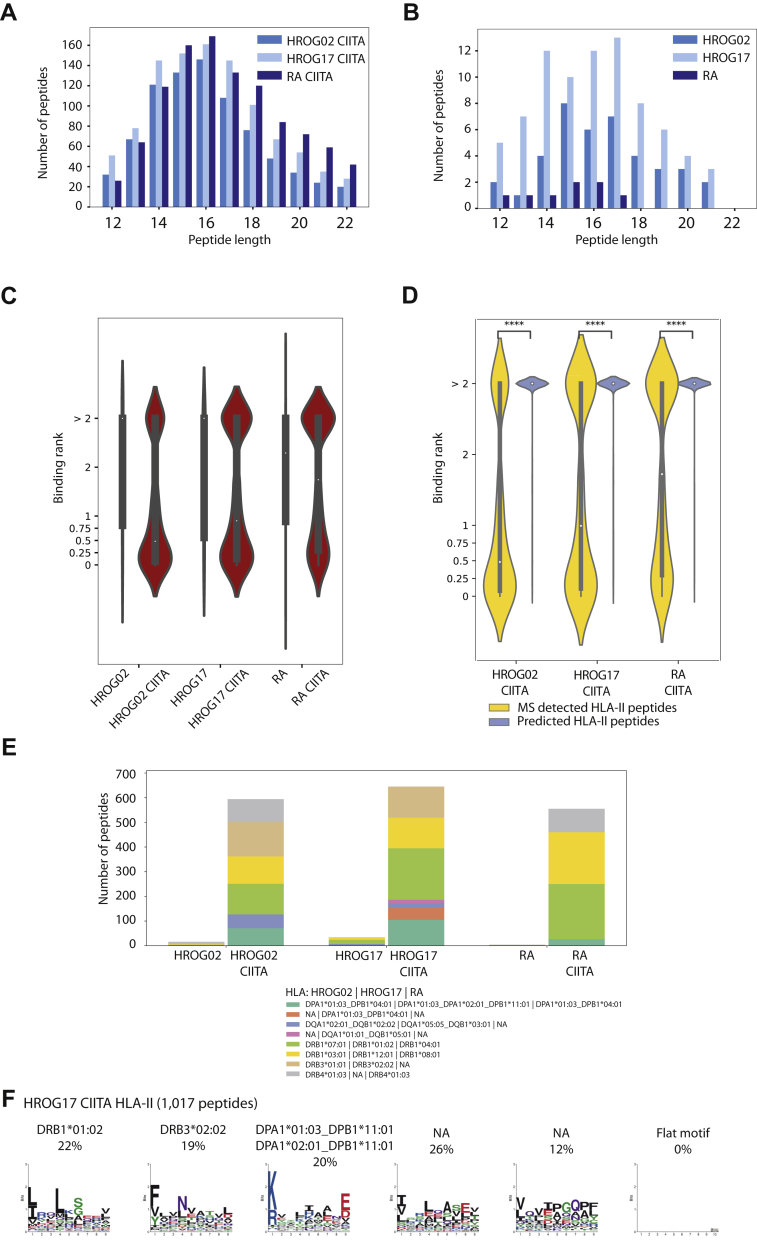
**MS-identified HLA-II peptides derived from exogenous bovine proteins that are included in the cell culture growth media.** Length distribution of HLA-II peptides derived from bovine proteins in HROG02-CIITA, HROG17-CIITA, and RA-CIITA (*A*) and HROG02, HROG17, and RA cells (*B*). Violin plots show the distribution of the predicted binding ranks of the identified bovine-derived peptides to the respective HLA class II alleles expressed in the different samples computed by MixMHC2pred (*C*). A comparison was made with a pool of *in silico* predicted 12 to 22 amino acids peptides from the same source bovine proteins (*D*), where all peptides identified by MS in any of the samples were removed. For each peptide, the HLA allele with the lowest rank was assigned as binder. Peptides with multiple binding alleles assigned were removed. The distributions of predicted percentage binding ranks of 809, 820, and 967 MS-detected bovine HLA-II peptides from HROG02-CIITA, HROG17-CIITA, and RA-CIITA cells, respectively, were compared with those of 485,949, 486,054, and 486,055 *in silico* sequences in HROG02-CIITA, HROG17-CIITA, and RA-CIITA samples, respectively. Statistical difference between the distributions was assessed using Mann–Whitney *U* test. Because of the big difference in the number of peptides in the different data sets, and to keep violin plots visible and appealing, the width of the violins is constant across the different samples and data sets. ∗∗∗∗*p*-value ≤ 1.00e−04. Distribution of predicted binders (rank ≤ 2%) to the respective alleles in each of the samples (*E*). Motif deconvolution of all 12 to 22 a.a HLA-II peptides identified in the HLA-II ligandome of HROG17-CIITA sample derived from bovine proteins (*F*). Sample name and number of peptides are indicated. Numbers above each motif indicate the percentage of peptides assigned to that motif. In case of observed multiple specificities, motifs are indicated with the same HLA allotype. NA, not assigned.

It has been reported before that CIITA-transfected tumor cells gained ability to prime *in vivo* naïve CD4+ cells, and thus, they may serve as APCs (19). We found 809, 1017, and 1048 peptides in the HLA-II peptidome of HROG02-CIITA, HROG17-CIITA, and RA-CIITA cells respectively, derived from presumably contaminant bovine proteins that are typically routinely included in growth media of human cells as serum protein supplements, including serum albumin, alpha-1-antiproteinase, hemopexin, Complement C3, Apolipoprotein A-I, and Alpha-2-HS-glycoprotein. Such exogenous peptides are typically excluded from downstream application. We found that the resulting peptides have a similar length distribution as HLA-II peptides derived from human source proteins (Fig. 6, *A* and *B*). In total, 68%, 63%, and 52% of these bovine-derived peptides are predicted to bind the respective HLA allotypes in HROG02-CIITA, HROG17-CIITA, and RA-CIITA cells (MixMHC2pred rank ≤2%) (Fig. 6*C*, supplemental Table S5). The repertoire of MS-detected peptides derived from bovine proteins was significantly enriched (*p* ≤ 1.00e−04, Mann–Whitney *U* test) in predicted HLA-II ligands, as compared with all other 12 to 22 mer peptide sequences in silico generated from the same bovine source proteins (485,949, 486,054, and 486,055 *in silico* peptides in HROG02-CIITA, HROG17-CIITA, and RA-CIITA samples, respectively), where sequences overlapping with the MS-detected peptidome were removed (Fig. 6*D*). In HROG17-CIITA, independent motif deconvolution analysis with MoDec revealed three motifs that showed similarity to the binding motifs of DRB1∗01:02, DRB3∗02:02, and DPA1∗01:03_DPB1∗11:01 and DPA1∗02:01_DPB1∗11:01. The distribution between the HLA-II allotypes is in agreement with the deconvoluted motifs that are less defined due to the relative low number of peptides available for this analysis (Fig. 6, *E* and *F*). Overall, this data suggests that GBM CIITA-dependent HLA-II positive cells have the ability to present exogenous antigens at least when expanded in culture. This hypothesis is supported by previous functional demonstration that other human tumor cell lines rendered HLA-II positive by transfection with CIITA process and present *in vitro* exogenously derived peptides (58).

### Unraveling a Large Repertoire of Tumor-Associated HLA-I and HLA-II Antigens in CIITA Expressing GBM Cells

We first performed a proteo-genomics analysis in the HROG02 and HROG17 cells in order to identify mutated neoantigens. We performed whole-exome sequencing on the cancer cells and matched blood cells, called nonsynonymous somatic mutations, and generated for each sample a personalized proteome reference that includes the private mutations. However, we could not identify any mutated neoantigens in these lines, presumably due to the low tumor mutation burden of 8.15 and 8.125 SM/Mb detected by two or more callers in HROG02 and HROG17 cells, respectively.

Next, we focused on the identification of peptides derived from shared TAAs. GBM cells typically do not express HLA-II molecules. As a result, very low number of HLA-II peptides have been identified before in MS immunopeptidomics studies, and not much is known about the landscape of TAAs that can be presented on HLA-II in GBM (9). Therefore, we first verified that known GBM-associated TAAs are presented on HLA-I molecules in our three investigated GBM lines. We compared the HLA-I antigenic repertoire with the repertoire reported in a recent study where a large-scale HLA-I related antigen discovery was performed in tens of GBM tissues and plasma samples (59). Shraibaman *et al.* (59) shortlisted 134 cancer-testis antigens (CTAs) of high clinical interest that are expressed at a very low level in healthy tissues from which they identified 77 HLA-I ligands belonging to 32 CTAs. We found 46 HLA-I peptides derived from 16 CTAs from this list. Five HLA-I peptides were common between the two studies, while we identified additional six peptides from the CTAs MAGEA1, MAGEA12, and paired box protein Pax-3 (PAX3) that were not found by Shraibaman *et al.* (supplemental Table S8). Importantly, we detected 179 HLA-II ligands from 12 of the CTAs, 114 of them derived from the heat shock 70 kDa protein 1A (HSPA1A), while others derived from additional CTAs such as Hepatocyte growth factor receptor (MET, 14 HLA-II peptides) and Macrophage colony-stimulating factor 1 (CSF1, 10 HLA-II peptides) (supplemental Table S9).

Next, we further identified in the HLA-I ligandome of the three cells lines 138 peptides derived from known GBM-associated tumor antigens that have been used as source proteins for a variety of GBM vaccines (Table 2 and supplemental Table S10). More specifically, we identified in the HLA-I ligandome of HROG02-CIITA cells two HLA-A∗02:01 immunogenic epitopes that have been included in the IMA950 peptide-based vaccine, TMLARASA from CSPG4, and KIQEILTQV from insulin-like growth factor 2 mRNA-binding protein 3 (IGF2BP3) and in HROG02 cells the AMTQLLAGV peptide from TNC (60,61). Additional confirmed immunogenic epitopes, specific for the well studies HLA-A∗02:01, HLA-A∗01:01, and HLA-A∗24:02 allotypes, were identified in the HLA-I ligandome of HROG02, HROG02-CIITA, RA, and RA-CIITA cells. These allotypes are expressed in HROG02 (HLA-A∗02:01 and HLA-A∗01:01) and RA cells (HLA-A∗02:01 and HLA-A∗24:02), and not in HROG17 cells, which can explain the lack of detection of immunogenic epitopes in the latter (Table 3).

**Table 2:** A list of known TAAs in GBM from which peptides have been used in vaccine trials in GBM patients

Gene name	Protein name	Number of HLA ligands from this study		Proteome expression level (quantile 1–4)	Vaccine name	Trial number	Reference (PMID)
		HLA-I	HLA-II				
Multiple genes	Multiple proteins	108	89		APVAC1	NCT02149225	30568303
BCAN ∗	Brevican core protein	0	0	ND	APVAC1; IMA950	NCT02149225; NCT01222221; NCT03665545	30568303; 27225692; 30753611
PTPRZ1 ∗	Receptor-type tyrosine-protein phosphatase zeta	0	0	ND	APVAC1; IMA950	NCT02149225; NCT01222221; NCT03665545	30568303; 27225692; 30753611
BIRC5 ∗	Baculoviral IAP repeat-containing protein 5	0	0	Q1	APVAC1; IMA950; SL-701	NCT02149225; NCT01222221; NCT02078648	30568303; 27225692; 30753611; PMC6216116
MET ∗	Hepatocyte growth factor receptor	3	14	Q2	IMA950	NCT01222221	27225692; 30753611
CSPG4	Chondroitin sulfate proteoglycan 4	8	73	Q4	IMA950	NCT01222221; NCT03665545	27225692; 30753611
FABP7	Fatty-acid-binding protein, brain	0	0	Q1	IMA950	NCT01222221; NCT03665545	27225692; 30753611
IGF2BP3	Insulin-like growth factor 2 mRNAbinding protein 3	2	1	Q4	IMA950	NCT01222221; NCT03665545	27225692; 30753611
NLGN4X	Neuroligin-4, X-linked	1	2	Q1	APVAC1; IMA950	NCT02149225; NCT01222221; NCT03665545	30568303; 27225692; 30753611
NRCAM	Neuronal cell adhesion molecule	0	5	Q1	APVAC1; IMA950	NCT02149225; NCT01222221; NCT03665545	30568303; 27225692; 30753611
TNC	Tenascin	8	20	Q4	IMA950	NCT01222221; NCT03665545	27225692; 30753611
ERBB2	Receptor tyrosine-protein kinase erbB-2	1	13	ND	ICT-107; Long peptide vaccine	NCT01280552; NCT02754362	22847020
DCT	L-dopachrome tautomerase	0	1	ND	ICT-107	NCT01280552	22847020
PMEL	Melanocyte protein PMEL	0	0	ND	ICT-107	NCT01280552	22847020
MAGEA1	Melanoma-associated antigen 1	3	0	Q3	ICT-107	NCT01280552	22847020
PROM1	Prominin-1	0	0	ND	ICT-121	NCT02049489	
IL13RA1	Interleukin-13 receptor subunit alpha-1	1	3	ND	ICT-107; Long peptide vaccine; SL-701	NCT01280552; NCT02754362; NCT02078648	22847020; PMC6216116
EGFRvIII	Epidermal growth factor receptor variant III	0	0	ND	Long peptide vaccine	NCT02754362	
EPHA2	Ephrin type-A receptor 2	1	50	Q4	Long peptide vaccine; SL-701	NCT02754362; NCT02078648	PMC6216116
CHI3L1	Chitinase-3-like protein 1	2	8	Q2	Long peptide vaccine	NCT02754362	
TERT ∗	Telomerase	0	0	ND	UCPVax-Glio	NCT04280848	23032748

ND, not detected.

For each TAA the total number of HLA-I and HLA-II peptides identified in this study and the average expression level of the source proteins across the three CIITA-expressing GBM cell lines are indicated. Average protein expression from shotgun proteomics is reported as quantile level. The asterisk highlights source proteins of HLA-II epitopes that were included in the vaccines.

**Table 3:** A list of MS-detected HLA-I peptides that were previously shown to be immunogenic in GBM patients

Gene name	Sequence	HLA restriction	Average MS intensity HLA-I peptides				Known immunogenic HLA-I epitopes in GBM		Reference (PMID)
			HROG02 _CIITA	HROG02	RA_CIITA	RA	Peptide-based vaccine trial	Immunogenicity assay	
CLU	KLFDSDPITVTV	HLA-A∗02:01			22.90	23.79		ELISPOT (IFNg)	30568303
CSPG4	TMLARLASA	HLA-A∗02:01	29.09				IMA950	ICS	22418738
								Tretramer	30753611
IGF2BP3	KIQEILTQV	HLA-A∗02:01	27.52			24.02	IMA950	ICS	22418738
								Tretramer	30753611
IL13RA2	LLDTNYNLFY	HLA-A∗01:01	22.94					ICS	29557506
ORMDL1	TLTNIIHNL	HLA-A∗02:01	25.79	24.60				ELISPOT (IFNg)	30568303
PCDHGC5	GLDPSSGAIHV	HLA-A∗02:01	26.34	24.76	22.78	22.14		ELISPOT (IFNg)	30568303
PJA2	RYQESLGNTVF	HLA-A∗24:02			23.98	23.01		ELISPOT (IFNg)	30568303
PLEKHA4	LLQDRLVSV	HLA-A∗02:01				22.54		ELISPOT (IFNg)	30568303
RAD54 B	SLYKGLLSV	HLA-A∗02:01			22.28			ELISPOT (IFNg)	30568303
SMC4	HYKPTPLYF	HLA-A∗24:02			27.02	27.01		ELISPOT (IFNg)	30568303
TNC	AMTQLLAGV	HLA-A∗02:01		28.83			IMA950	ICS	22418738
								Tretramer	30753611
ZNF3	KYNDFGNSF	HLA-A∗24:02			23.73			ELISPOT (IFNg)	30568303

HLA restriction is indicated as well as Log2 transformed intensity of the MS-based detection in HROG02, HROG02CIITA, RA, and RA-CIITA samples. No such peptides were identified in HROG17 and HROG17-CIITA samples. Information on the inclusion of the epitopes in GBM peptide-vaccines and the method by which their immunogenicity was assessed in previous studies is provided.

Information on immunogenic HLA-II peptides in GBM is very limited, and among the few immunogenic HLA-II epitopes reported so far, that have been included in the GAPVAC and IMA950 trials, are from brevican core protein (BCAN), Survivin (BIRC5), Receptor-type tyrosine-protein phosphatase zeta (PTPRZ1), and MET. We identified in the HLA-II ligandome data sets of the stably expressing CIITA cells in total 279 peptides derived from known GBM-associated tumor antigens that have been used as source proteins for short or long-peptide-based vaccines tested on GBM patients (Table 2 and supplemental Table S11). The most prevalent source proteins were CSPG4, ephrin tape-A receptor 2 (EPHA2), TNC, and MET, from which 73, 50, 20, and 14 unique HLA-II peptides were identified, respectively. Overall, the TAAs from which we identified tens of HLA-II peptides were found to be abundantly expressed in the proteome of the CIITA-expressing GBM cells. Furthermore, most of the TAAs not detected in our immunopeptidomics data were also not found to be expressed (Table 2). In the non-CIITA transduced cells, only three of the 279 GBM-related TAA-peptides were identified.

## Discussion

Priming and activation of CD4+ cells are crucial for inducing and maintaining effective anticancer adaptive immune response. Hence, the identification of HLA-II cancer-specific epitopes is key to the development of potent cancer immunotherapies. In many solid tumors, and especially in GBM, HLA-II complexes are hardly ever naturally expressed on the surface of the transformed cells, though they can be induced to some extent under inflamed conditions. Hence, the identification of cancer-associated HLA-II epitopes requires laborious and rather artificial *in vitro* screening methods. So far, little is known about immunogenic HLA-II epitopes in GBM. Through stable expression of CIITA in three GBM cell lines coupled to a detailed and sensitive MS-based immunopeptidomics analysis, we here uncovered a remarkable breadth of the HLA-II ligandome in GBM. Like IFNɣ treatment, this approach leverages and engages the natural cellular machinery of the cells, yet without drastically affecting the HLA-I ligandome or other cellular pathways.

HLA binding prediction and binding motif deconvolution approaches revealed that the resulting presented HLA-II repertoire is rich, comprising ligands of the HLA-DR, -DP, and -DQ molecules. In recent years, the performance of HLA-I and HLA-II peptide binding prediction tools has greatly improved mainly through the inclusion of large training data sets of MS-detected HLA ligands either from mono-HLA-allelic cell lines or from mixed HLA alleles following motif deconvolution (51,55,62, 63, 64, 65, 66). A common bias in MS-trained predictors is the depletion of cysteine-containing peptides in the training data sets, though it can be corrected to some degree by expanding the MS spectral search to include modified cysteine residues (67) or with post search renormalization of the position weight matrix based on observed amino acid frequencies at nonanchor positions (49). Furthermore, due to the lower expression level of HLA-DQ and -DP molecules, their representation in deconvoluted MS-ligandomic data is limited, and hence less training data is available for DQ and DP and especially for rare alleles. While the majority of the HLA-II allelic combinations expressed in the three GBM CIITA-expressing cell lines are covered by the MixMHC2pred, the allelic coverage for HLA-DQ and –DP is lower than, for example, abundant and prevalent HLA-DR alleles (51). More advanced HLA-pan predictors, such as the NetMHCIIpan (55), can predict binding for HLA allotypes for which no direct ligandomic or *in vitro* binding training data is available; nevertheless, the allelic coverage is also not complete and prediction accuracy of panpredictors can be suboptimal (63). Indeed, in HR0G02 and HROG17 CIITA-expressing cells, more than 80% of the identified peptides were predicted to bind any of the expressed HLA-II allotypes, most of them were assigned to HLA-DR allotypes. Only 70% of the peptides were predicted to be binders in RA-CIITA cells; however, here, rare HLA-DP alleles (HLA-DPA1∗02:07_DPB1∗04:01 and HLA-DPA1∗02:07_DPB1∗19:01) are expressed that are currently not covered by both NetMHCIIpan and MixMHC2pred predictors.

Is has been demonstrated that, when the performance of HLA ligand predictors is suboptimal, binding motif deconvolution of in-depth MS immunopeptidomics data may assure that the eluted peptides of a given sample are indeed HLA ligands (68). We revealed with this complementary approach HLA-II binding motifs that were highly similar to known motifs, such as those defined by the NetMHCIIpan tool (55). The proportion of the assigned peptides to each motif was in agreement with the prediction results obtained by MixMHC2pred. Importantly, when redundant motifs occur in a sample, as in the case of the HLA-DQ alleles in HROG02-CIITA cells, unbiased clustering is typically not capable of resolving each of the four DQA1 and DQB1 combinations. Nevertheless, when HLA-II motifs are so much alike, also binding predictors will output highly similar prediction scores. In-depth MS ligandomic data of cells uniquely expressing each of the above HLA-II allelic combination could reveal nuances in their binding specificities and binding modes (56), and such data is expected to improve the performance of current predictors and to increase their allelic coverage.

Next, we further extended our investigation to explore characteristics of the proteins that are sampled for presentation in the CIITA-expressing GBM cells. Our data suggest that CIITA expression in GBM cells seems to not interfere with sampling of the proteome for HLA-I presentation. Consistent with results from a previous study (57), we found that the source proteins of HLA-II peptides are commonly derived from self-endogenous proteins located in the vesicular compartments, lysosomes, and phagosomes and are involved in particular cellular processes such as signal transduction and cell adhesion (69,70). In contrast, source proteins involved in transcription, DNA maintenance, spliceosome, cell cycle, and transcription factors are less often presented on HLA-II, but are frequently presented and enriched on HLA-I molecules. Importantly, we found high similarity between the cellular compartments and pathways that are sampled for HLA-II presentation in CIITA-expressing GBM cells and B and T cells that naturally express HLA-II.

Our data indicate that CIITA-expressing GBM cells can also directly present external antigens as HLA-II ligands. We identified hundreds of peptides in each of the cell lines, derived from bovine proteins that are abundant in serum-supplemented culture media. Presentation of such bovine serum proteins is common in APCs and has been shown already in the seminal work of Chicz and colleagues who characterized the HLA-DR ligandome of B cells in the early 90’s (69). In hepatocarcinoma cells, that like GBM cells are normally HLA-II negative but can express similarly high level of HLA-II upon CIITA transfection, it has been shown that CIITA-transduced cells were capable of processing the exogenously loaded *Mycobacterium tuberculosis* Ag85 protein to present a specific epitope recognized by an HLA-DR-restricted epitope-specific T cell line (58). Thus, CIITA-mediated induction of components of the HLA-II presentation machinery seems to equip the cells with the function of processing exogenous protein and presenting relevant peptides to CD4+ T cells. It will be interesting to assess the general validity of this approach and determine whether professional APCs and CIITA-transformed non-APCs, such as tumor cells, use the same basic machinery to process endogenous peptides. Within this frame, it will also be relevant to assess similarities and distinctions of the presented ligandomes and, specifically, the overlap in terms of presentation of immunogenic epitopes. In the case of tumors, would this phenomenon bear importance for the biology of HLA-II-expressing cancer cells, for example, in inflamed tumors? More research using this approach will shed light on such fundamental key questions.

While not much is known about TAA-derived HLA-II ligands in GBM, the repertoire of HLA-I ligands has been more intensively explored in recent years (9, 59, 60), and consequently, more immunogenic GBM related HLA-I epitopes are known and available for translational research and clinical applications. We confirmed that TAAs can be presented in the three CIITA-expressing GBM cell lines, and in total, 138 HLA-I peptides derived from known GBM-associated tumor antigens could be identified. For examples, we identified in HROG02 cells three HLA-A∗02:01 immunogenic epitopes that have been included in the IMA950 vaccine, TMLARASA from CDPG4, KIQEILTQV from IGF2BP3, and AMTQLLAGV from TNC (60, 61). Importantly, we identified 279 HLA-II peptides that have not yet been reported to be presented on GBM cells that are derived from known GBM-associated tumor-antigens that have been used previously as sources for short or long-peptide-based vaccine trials in GBM (23, 24, 25, 61, 71). If shown to be immunogenic, novel immunotherapeutic approaches could be developed in the future to target them.

GBM cells that stably express CIITA are decorated with remarkable high levels of HLA-II complexes. This renders these cells very attractive for antigen discovery endeavors, but more important, such engineered cells may have great therapeutic potential in clinical setting. For example, viral vectors containing expressable CIITA, alone or in association with oncolytic viruses (agents that can overcome blood–brain barrier), could be used to target established GBM tumors and make them more immunogenic for triggering and/or increasing the stimulation of tumor-specific TH cells (72). Preclinical vaccination research *in vivo* with tumor cells expressing CIITA led to an immune response against the CIITA-transfected tumor and, most importantly, against the parental tumor (17, 18). Vaccination with CIITA-driven MHC-II-expressing tumor cells has a potential to induce a potent TH immune response through a diverse antigenic repertoire and to transform the tumor microenvironment from a noninflamed to an inflamed phenotype (20). Engaging the endogenous immune system in combination with checkpoint blockers could potentially mount a broad and clinically effective response.

## Data Availability

MS raw files, MaxQuant proteomics, and immunopeptidomics output results have been deposited to the ProteomeXchange Consortium *via* the PRIDE (73) partner repository with the data set identifier PXD020079.

## Conflicts of interest

The authors declare no competing interests.
