# Supplementary material for: CIITA-Transduced Glioblastoma Cells Uncover a Rich Repertoire of Clinically Relevant Tumor-Associated HLA-II Antigens
Source: Mol Cell Proteomics. 2021 Jan 6;20:100032. doi: 10.1074/mcp.RA120.002201 (PMC8724627; doi:10.1074/mcp.RA120.002201)
Supplement: Supplementary Information and Figures [file mmc12.pdf]

Supplementary information

CIITA-transduced glioblastoma cells uncover a rich repertoire of clinically relevant tumor-associated HLA-II antigens

Greta Forlani[1#], Justine Michaux[2,3#], HuiSong Pak[2,3], Florian Huber[2,3], Elodie Lauret Marie Joseph[2,3], Elise Ramia[1], Brian J. Stevenson[4], Michael Linnebacher[5], Roberto Accolla[1]* and Michal Bassani-Sternberg[2,3]*

Supplementary Table 1: List of MS files of HLA immunopeptidomics samples and number of cells used for each replicate.

Supplementary Table 2: List of 6,245 proteins identified and quantified through shotgun proteomics in HROG02, HROG17 and RA cells with and without IFNɣ treatment and in HROG02-CIITA, HROG17-CIITA and RA-CIITA cells. The list is the result of filtering all identified proteins to include proteins with 3 valid LFQ intensity values in at least one group (cell line and condition). Intensity values were Log2 transformed.

Supplementary Table 3: List of HLA-I peptide sequences of length 8-12 amino acids identified in HROG02, HROG17, RA, HROG20-CIITA, HROG17CIITA and RA-CIITA samples, including mass and identification scores.

Supplementary Table 4: List of HLA-II peptide sequences of length 12-22 amino acids identified in HROG02, HROG17, RA, HROG02-CIITA, HROG17-CIITA and RA-CIITA samples, including mass and identification score.

Supplementary Table 5: MixMHCpred and MixMHC2pred prediction scores for all identified peptides, per sample, including peptides derived from bovine proteins.

Supplementary Table 6: Output from DAVID concerning GO annotation enrichment of biological process, molecular functions, cellular compartments and KEGG pathways for source proteins presented on HLA-I and on HLA-II complexes, in HROG02-CIITA, HROG17-CIITA and RA-CIITA cells, in the B cells lines CD165, CM647 and RA957 and in the expanded T cell cultures TIL1 and TIL3.

Supplementary Table 7: Lists of source proteins and the number of presented HLA-I and HLA-II peptides per sample used as input for DAVID and Proteomaps tool.

Supplementary Table 8: List of HLA-I peptides derived from the list of 134 CTAs from Shraibman et al.

Supplementary Table 9: List of HLA-II peptides derived from the list of 134 CTAs from Shraibman et al.

Supplementary Table 10: List of HLA-I peptides identified from source proteins of peptides used in GBM cancer vaccines.

Supplementary Table 11: List of HLA-II peptides identified from source proteins of peptides used in GBM cancer vaccines.

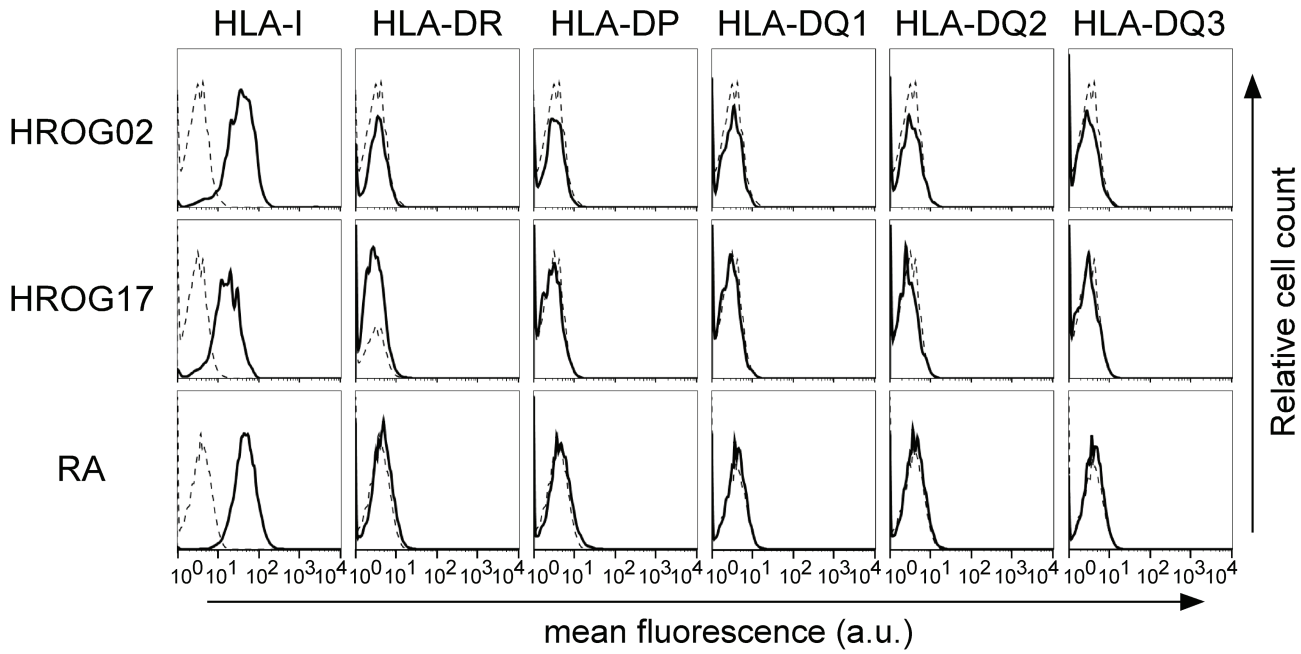

Supplementary Figure 1: HROG02, HROG17 primary GBM cells and RA GBM cell line do not express HLA-II molecules. HLA-I and HLA-II phenotyping of HROG02, HROG17 and RA cells was carried out by immunofluorescence and FACS analysis. Histograms represent fluorescence profiles of the cells indicated on the left, incubated with specific anti HLA-I common (HLA-A,B,C; B9.12.1) or HLA-II DR (D1.12), DP (B7/21), or DQ (BT/3.4; XIII358.4 and XIV466.2) mAbs (solid line), or with FITC-conjugated F(ab)2 anti-mouse antibody (dashed line). Mean fluorescence values are expressed in the abscissa as arbitrary units (a.u.).

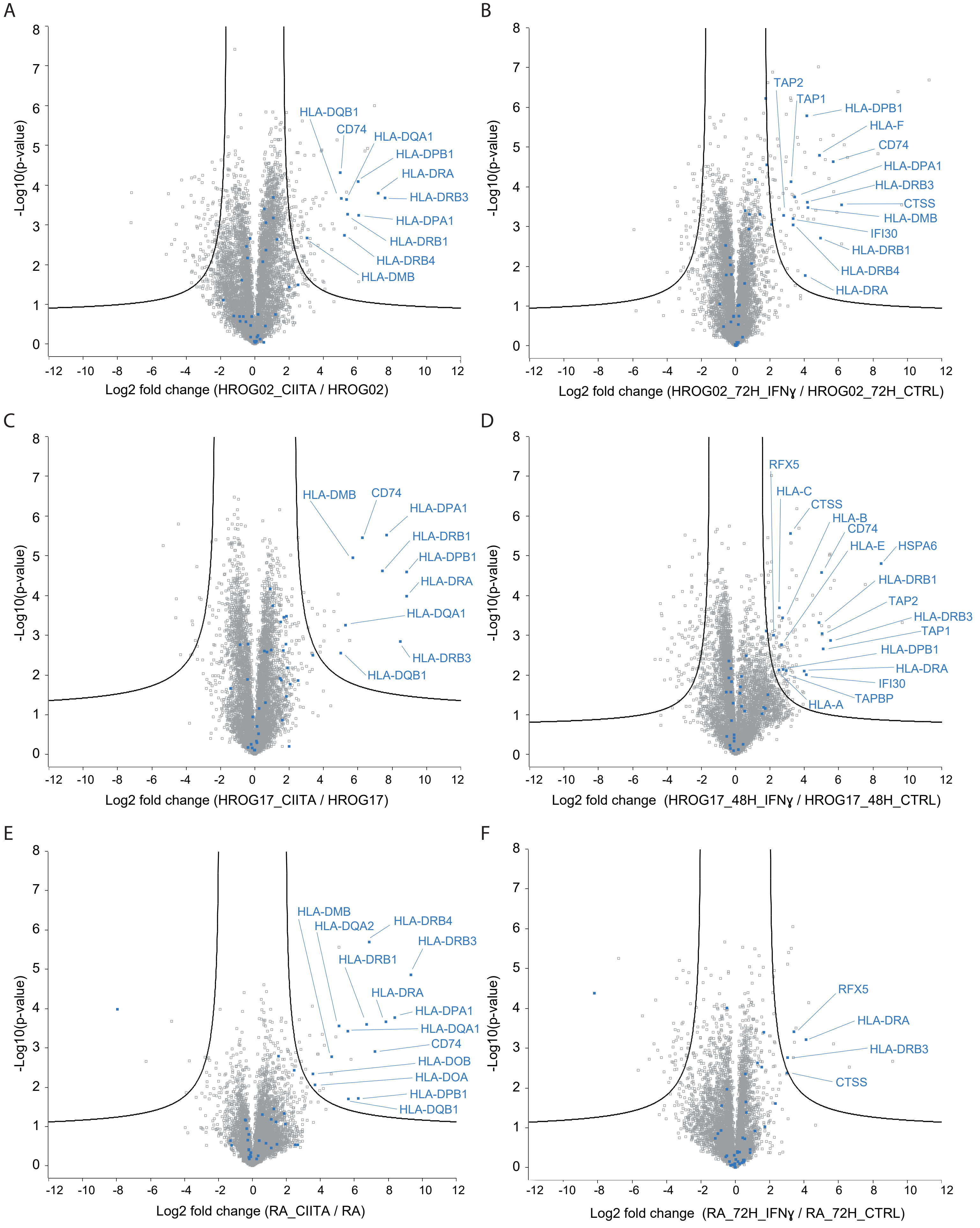

Supplementary Figure 2: Differential protein expression of HLA-I and HLA-II presentation machineries related proteins upon IFNγ treatment and stable expression of CIITA in HROG02 (A-B), HROG17 (C-D) and RA (E-F) cells. Volcano plots summarizing two-sided Student's t test results of 3 biological replicates per condition. Points outside the lines indicate significantly upregulated or downregulated proteins (permutation-based FDR=1%. S0=1). Proteins related to the 'antigen processing and presentation' pathway at the Kyoto Encyclopedia of Genes and Genomes (KEGG) database are highlighted in blue and those that are significantly upregulated are named.

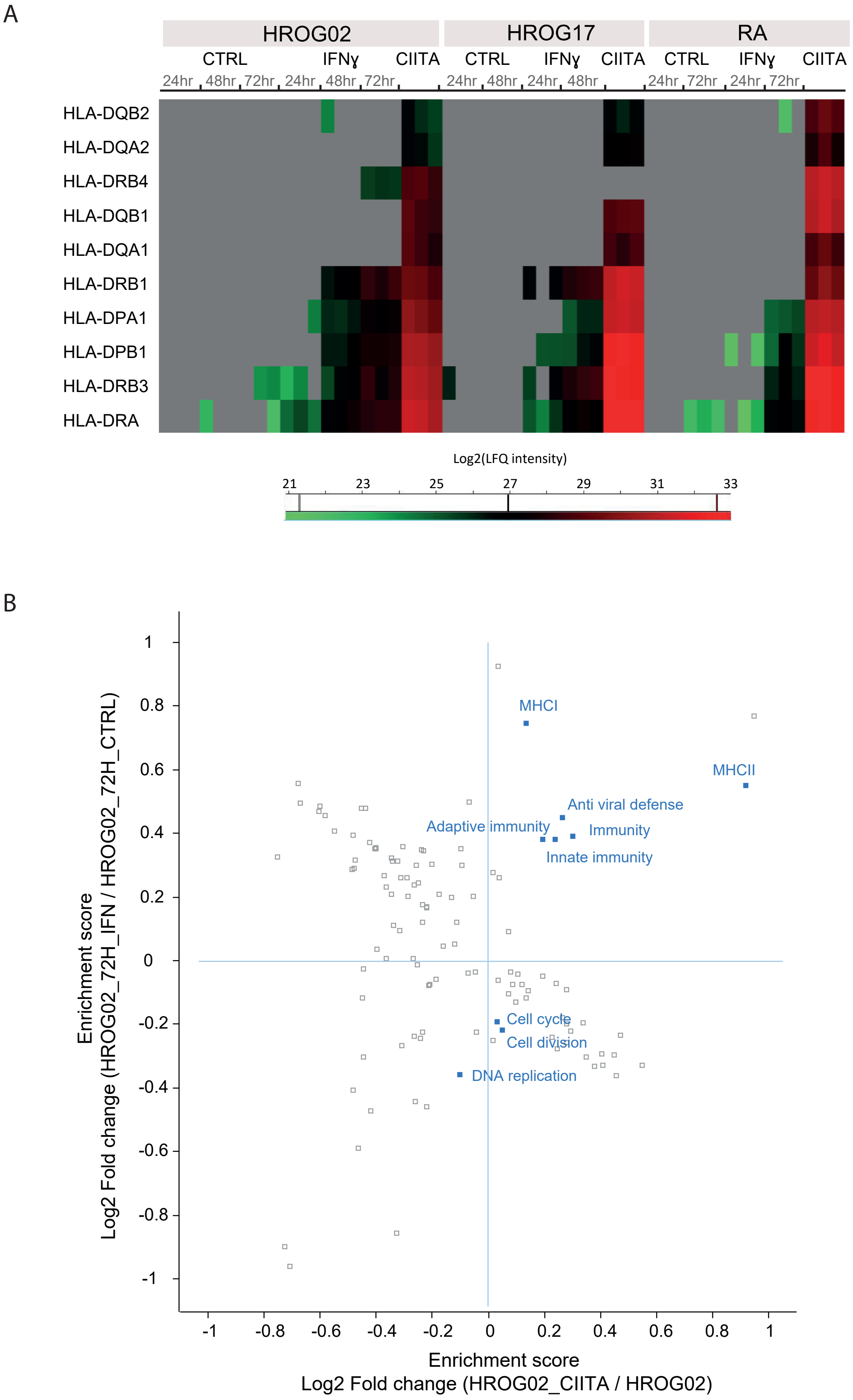

Supplementary Figure 3: Label free quantification of expression of HLA-II proteins upon treatment with IFNγ or stable expression of CIITA in HROG02, HROG17 and RA cells (A). Heat map of Log2 transformed label free quantification (LFQ) values defined by shotgun proteomic analyses of 3 biological replicates per condition without imputation of missing values. 2D annotation enrichment analysis (B) of differentially expressed proteins that are CIITA-induced (x axis) versus 72 hours IFNγ treatment-induced (y axis) in HROG02 cells (Benjamin-Hochberg 0.02 FDR).

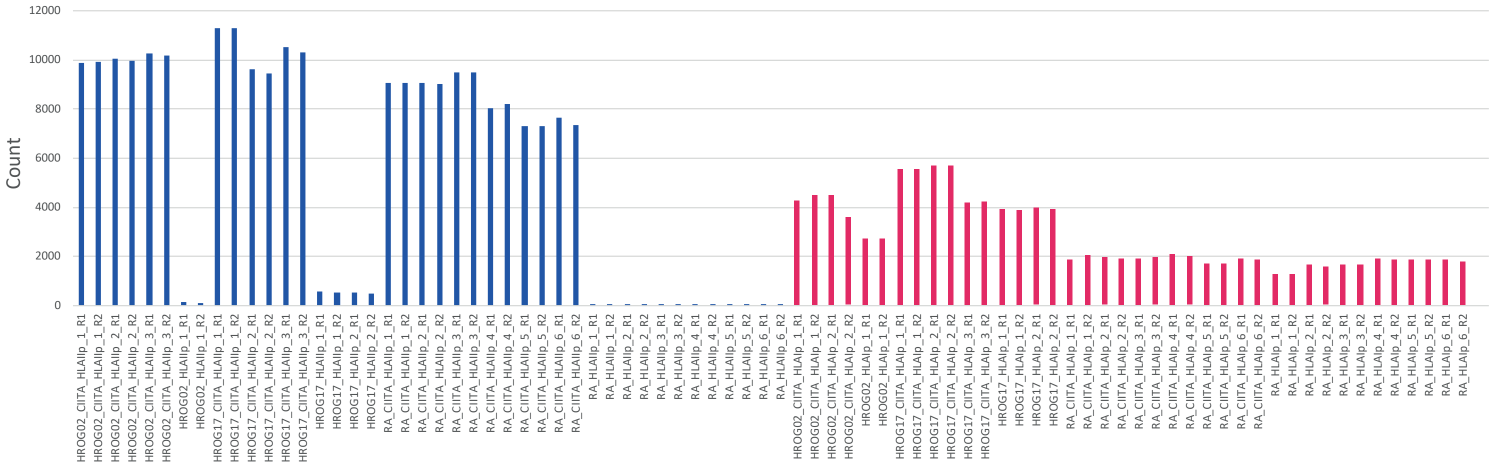

Supplementary Figure 4: Summary of number of identified 8-12 mer HLA-I peptides and 12-22 mer HLA-II peptides in each of the measured immunopeptidomics replicates.

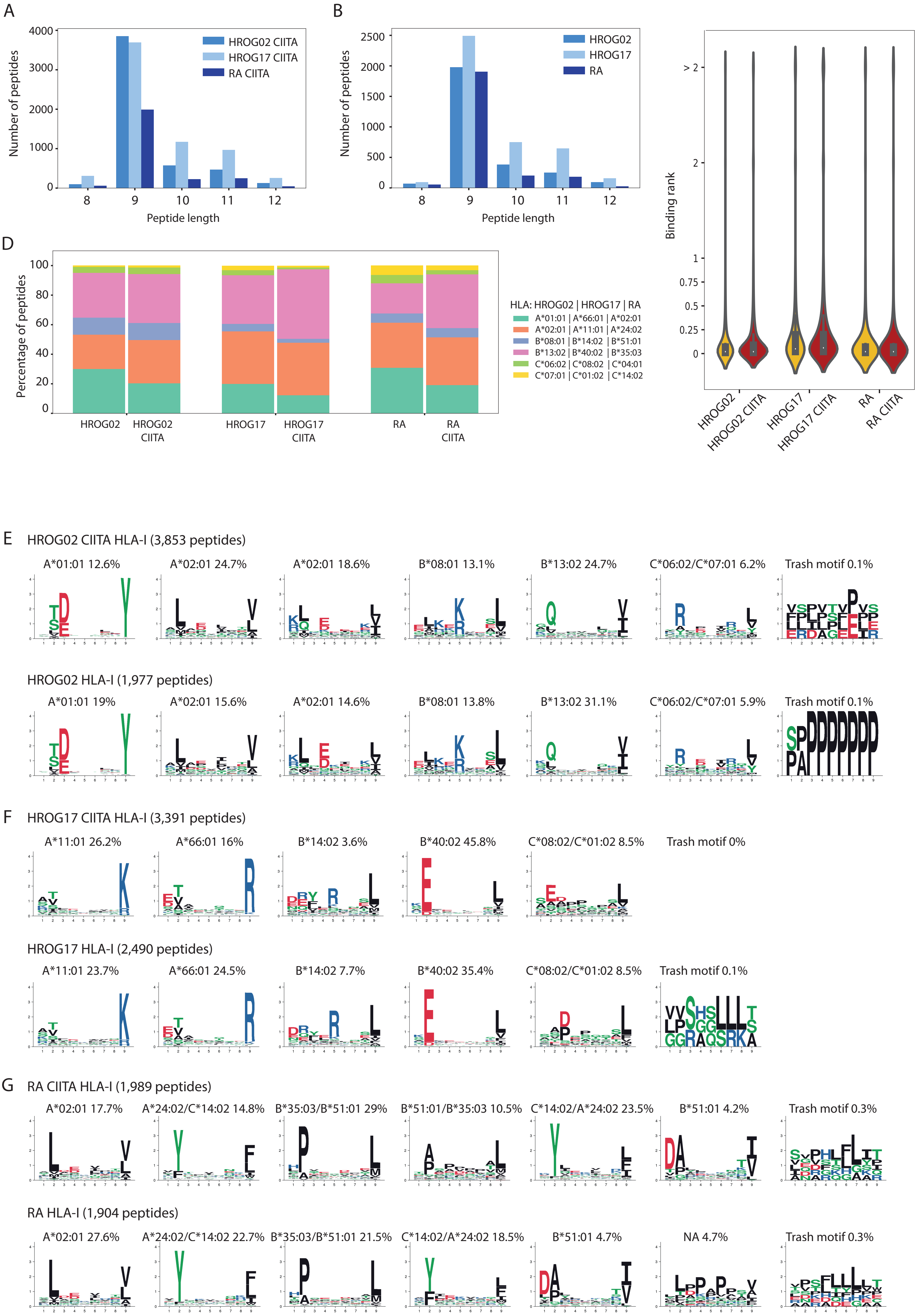
A
Number of peptides
Peptide length
HROG02 CIITA
HROG17 CIITA
RA CIITA
B
Number of peptides
Peptide length
HROG02
HROG17
RA
Binding rank
> 2
HROG02
HROG02 CIITA
HROG17
HROG17 CIITA
RA
RA CIITA
D
Percentage of peptides
HLA: HROG02 | HROG17 | RA
A*01:01 | A*66:01 | A*02:01
A*02:01 | A*11:01 | A*24:02
B*08:01 | B*14:02 | B*51:01
B*13:02 | B*40:02 | B*35:03
C*06:02 | C*08:02 | C*04:01
C*07:01 | C*01:02 | C*14:02
HROG02
HROG02 CIITA
HROG17
HROG17 CIITA
RA
RA CIITA
E HROG02 CIITA HLA-I (3,853 peptides)
A*01:01 12.6%
A*02:01 24.7%
A*02:01 18.6%
B*08:01 13.1%
B*13:02 24.7%
C*06:02/C*07:01 6.2%
Trash motif 0.1%
HROG02 HLA-I (1,977 peptides)
A*01:01 19%
A*02:01 15.6%
A*02:01 14.6%
B*08:01 13.8%
B*13:02 31.1%
C*06:02/C*07:01 5.9%
Trash motif 0.1%
F HROG17 CIITA HLA-I (3,391 peptides)
A*11:01 26.2%
A*66:01 16%
B*14:02 3.6%
B*40:02 45.8%
C*08:02/C*01:02 8.5%
Trash motif 0%
HROG17 HLA-I (2,490 peptides)
A*11:01 23.7%
A*66:01 24.5%
B*14:02 7.7%
B*40:02 35.4%
C*08:02/C*01:02 8.5%
Trash motif 0.1%
G RA CIITA HLA-I (1,989 peptides)
A*02:01 17.7%
A*24:02/C*14:02 14.8%
B*35:03/B*51:01 29%
B*51:01/B*35:03 10.5%
C*14:02/A*24:02 23.5%
B*51:01 4.2%
Trash motif 0.3%
RA HLA-I (1,904 peptides)
A*02:01 27.6%
A*24:02/C*14:02 22.7%
B*35:03/B*51:01 21.5%
C*14:02/A*24:02 18.5%
B*51:01 4.7%
NA 4.7%
Trash motif 0.3%

Supplementary Figure 5: Characterization of MS-identified HLA-I peptides. Length distribution of HLA-I peptides identified in HROG02-CIITA, HROG17-CIITA and RA-CIITA (A) and HROG02, HROG17 and RA (B) cells. Violin plots show the distribution of the predicted percentage binding ranks to the respective HLA class I alleles expressed in the different samples computed by MixMHCpred (C). Distribution of predicted binders (rank ≤ 2%) to the respective alleles in each of the samples (D). Motif deconvolution of all 9 mer HLA-I peptides identified in the ligandome of HROG02-CIITA and HROG02 (E), HROG17-CIITA and HROG17 (F), and RA-CIITA and RA (G) samples. Sample name and number of peptides are indicated. Numbers above each motif indicate the percentage of peptides assigned to that motif. NA; not assigned.

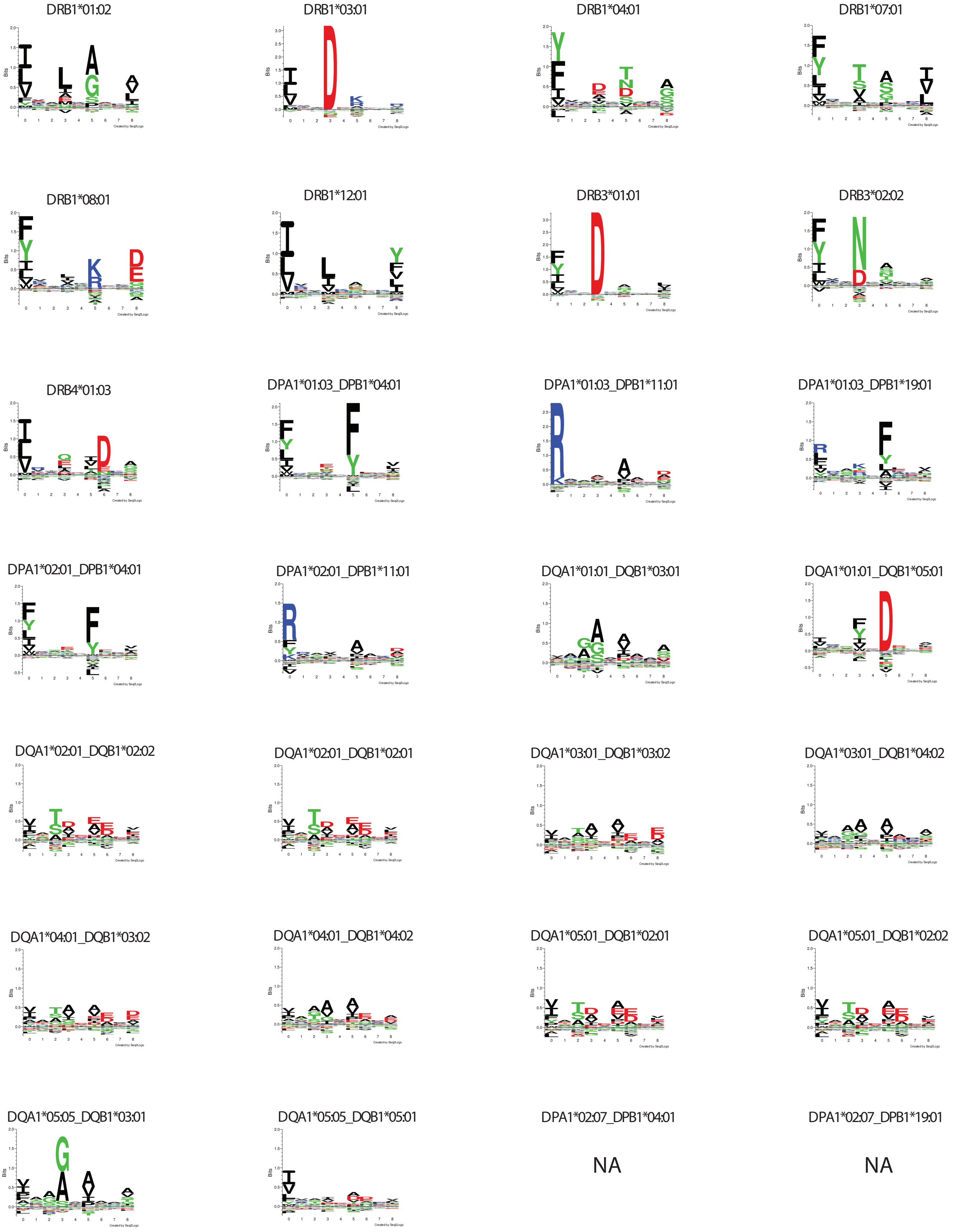

Supplementary Figure 6: Reference HLA-II binding motifs from NetMHCIIpan (Reynisson et al.) of HLA-II allotypes expressed in the investigated cells. NA- not available.

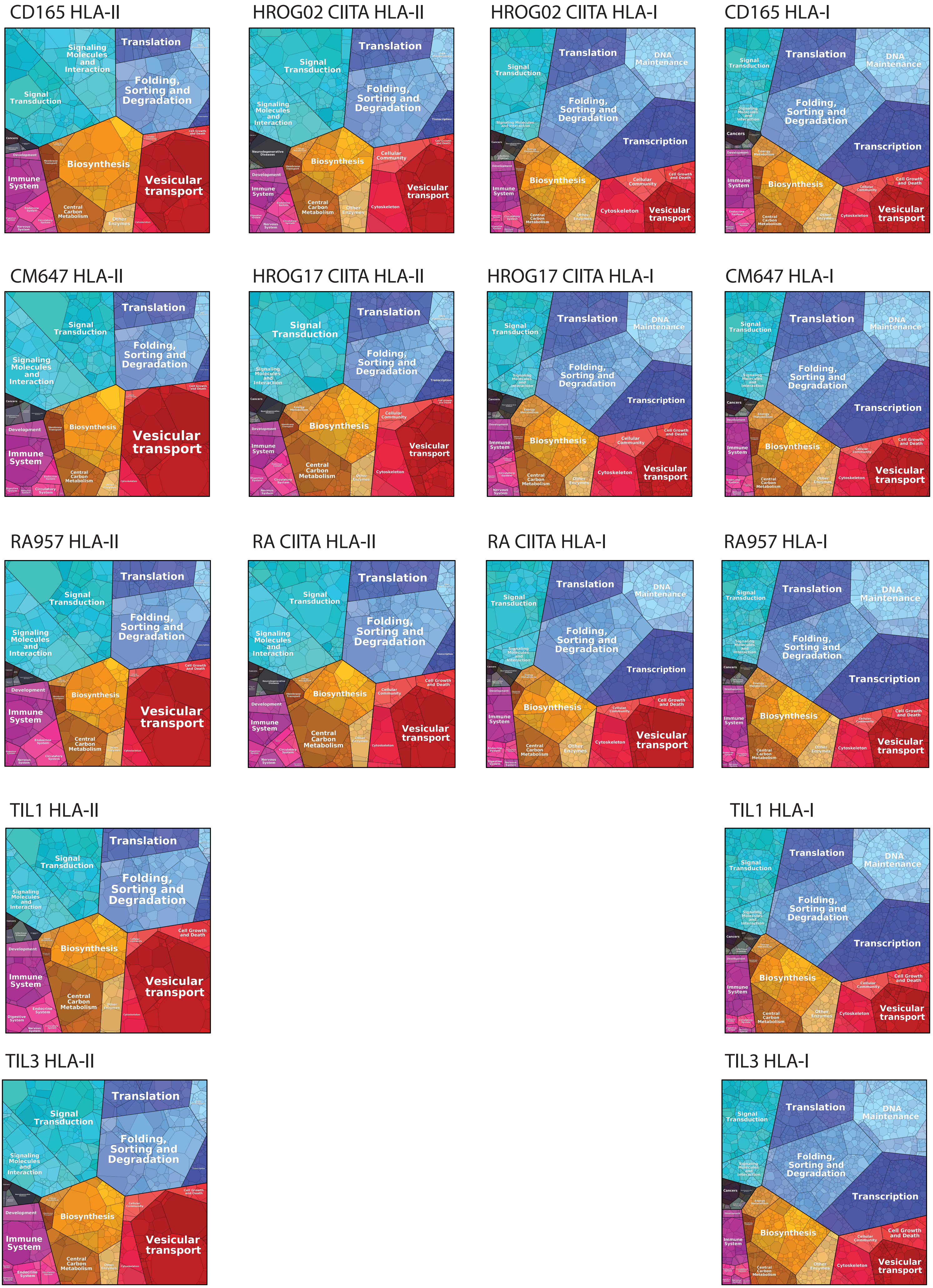
CD165 HLA-II
HROG02 CIITA HLA-II
HROG02 CIITA HLA-I
CD165 HLA-I
CM647 HLA-II
HROG17 CIITA HLA-II
HROG17 CIITA HLA-I
CM647 HLA-I
RA957 HLA-II
RA CIITA HLA-II
RA CIITA HLA-I
RA957 HLA-I
TIL1 HLA-II
TIL1 HLA-I
TIL3 HLA-II
TIL3 HLA-I

Supplementary Figure 7: Proteomaps visualization of the level of presentation of source proteins presented on HLA-I and on HLA-II complexes, in HROG02-CIITA, HROG17-CIITA and RA-CIITA cells, in the B cells lines CD165, CM647 and RA957 and in the expanded T cell cultures TIL1 and TIL3. The maps show quantitative composition of the presented proteomes that are arranged in multiple levels. Each protein is represented by a polygon, whose area reflects the number of peptides weighted by protein size. Proteins are classified according to their cellular function.
